# The Nodding syndrome cerebrospinal fluid proteome: a lens into neurodevelopmental failure consistent with environmentally triggered *MECP2* dysregulation?

**DOI:** 10.3389/fnmol.2026.1717920

**Published:** 2026-01-29

**Authors:** Raquel Valdes Angues, Caesar Okot, Keith D. Zientek, Phillip A. Wilmarth, Ashok P. Reddy, Alfred Lucid Blair Odong, Valerie S. Palmer, Lucy Kipwola Abwola, Ensio Ogal, Geoffrey Okello, Peter S. Spencer

**Affiliations:** 1 Department of Neurology, School of Medicine, Oregon Health & Science University, Portland, OR, United States; 2 Caseworker in Gulu and Kitgum, Gulu, Uganda; 3 Proteomics Shared Resources, Oregon Health & Science University, Portland, OR, United States; 4 Kitgum General Hospital, Kitgum, Uganda; 5 Department of Neurology, School of Medicine, and Oregon Institute of Occupational Health Sciences, Oregon Health & Science University, Portland, OR, United States

**Keywords:** biotoxins, CSF proteomics, epilepsy, MeCP2, MIA, neurodevelopment, Nodding syndrome, tauopathy

## Abstract

**Introduction:**

Nodding Syndrome (NS) is a childhood-onset epileptic encephalopathy of unknown etiology, occurring in clustered outbreaks across East Africa. Despite extensive investigation, its molecular underpinnings remain unresolved.

**Methods:**

We performed an 18-plex tandem mass tag (TMT)-based quantitative proteomic analysis of immunodepleted cerebrospinal fluid (CSF) from Ugandan NS patients (*n* = 9) and age-comparable Ugandan Controls (*n* = 9). Differential protein abundance and pathway-level enrichment analyses were conducted to identify dysregulated molecular networks.

**Results:**

A total of 2,195 CSF proteins were quantified, of which 544 showed statistically significant differential abundance. Dysregulated pathways spanned immune signaling, proteostasis, synaptic function, metabolism, transcriptional regulation, neurovascular integrity, and tau-associated processes. Notably, the NS CSF proteomic profile showed substantial pathway-level convergence with that reported in *MECP2* duplication syndrome (MDS), an X-linked neurodevelopmental disorder marked by *MECP2* overexpression and systemic immune-metabolic dysfunction. Clinically, NS shares features with both MDS and its mechanistic converse, Rett syndrome, characterized by *MECP2* loss-of-function.

**Discussion:**

These convergent molecular and clinical signatures suggest that NS may involve aberrant regulation of *MECP2*-associated networks. We propose a provisional model in which NS represents an environmentally induced functional phenocopy of *MECP2* network dysregulation, shaped by early-life immune and epigenetic perturbations and amplified by postnatal environmental stressors. Although direct epigenetic data and detailed exposure histories are currently limited, this integrative framework provides a testable model linking proteomic alterations and clinical observations to neurodevelopmental and immune-metabolic mechanisms, offering tractable directions for future mechanistic and therapeutic inquiry.

## 1 Introduction

Nodding syndrome (NS) is a devastating pediatric brain disorder that has emerged in epidemic clusters across East Africa (Metanmo et al., 2024). Onset between ages 3 and 18 is defined by repetitive head drops from atonic seizures that progress to epilepsy, cognitive, behavioral, and motor decline, sometimes with parkinsonian features (Dowell et al., 2013; Idro et al., 2013a,b; Spencer et al., 2013a,b, 2019). Brain pathology consistently reveals microglial activation and tauopathy (notably in the neocortex and locus coeruleus, and frequently in the substantia nigra and tegmental nuclei), but whether neuroinflammation drives or follows neurodegeneration is debated (Pollanen et al., 2018, 2023; Hotterbeekx et al., 2019). Beyond the brain, NS presents as a multisystem disorder, with frequent stunting, delayed puberty, musculoskeletal anomalies, and recurrent infections (Winkler et al., 2008; Idro et al., 2018; Piloya-Were et al., 2014; Valdes Angues et al., 2022; Abd-Elfarag et al., 2021; Ogwang et al., 2021; Edridge et al., 2023).

Despite decades of investigation, the etiology of NS remains unresolved (Foltz et al., 2013; Spencer et al., 2022; Korevaar and Visser, 2013; Olum et al., 2020; Spencer et al., 2017, 2016). Early reports of co-infection with *Onchocerca volvulus* (OV) and *Mansonella perstans* (MP) (Spencer et al., 2013a) fueled parasitic and autoimmune hypotheses (Colebunders et al., 2014, 2016a,b; Johnson et al., 2017; Idro et al., 2016). However, children in early disease stages (< 1 year after symptom onset) have tested positive for MP and *Necator americanus* (NA) but not for OV, suggesting a broader, multi-pathogen exposure profile (Edridge et al., 2023). Case-control studies from northern Uganda also linked symptom onset to the ingestion of moldy maize, a common source of mycotoxins (Spencer et al., 2016; Spencer, 2023), while ecological evidence highlights the potential involvement of exposure to cyanobacteria-rich stagnant waters that support the midges transmitting MP (Spencer et al., 2024). Together, these findings implicate biotoxins, namely cyanotoxins and mycotoxins, as candidate environmental triggers of NS.

We recently advanced the hypothesis that NS may reflect biotoxin-driven dysregulation of methyl-CpG binding protein 2 (MECP2) (Spencer et al., 2024), a dosage-sensitive epigenetic regulator of neurodevelopment, immune balance, and tau homeostasis (Guy et al., 2011; Tillotson and Bird, 2020; Maphis et al., 2017; Nan et al., 1997; Connolly and Zhou, 2019; Gulmez Karaca et al., 2019; Piccolo et al., 2019). Untargeted cerebrospinal fluid (CSF) proteomics, reported here for the first time, lends molecular support for this model, revealing convergent dysfunction across immune, proteostatic, synaptic, metabolic, transcriptional, and neurovascular domains, consistent with progressive network desynchronization. This proteomic profile mirrors, to a considerable extent, that of *MECP2* duplication syndrome (MDS), an X-linked disorder caused by *MECP2* overexpression and characterized by transcriptional silencing, immunodeficiency, and tauopathy (Cronk et al., 2017; Montgomery et al., 2018). Other clinical and proteomic features of NS echo those of Rett syndrome, a genetic disorder primarily associated with *MECP2* loss-of-function (Vilvarajan et al., 2023). These syndromic parallels support a central principle, namely that disruption of *MECP2* homeostasis, whether through excess, deficiency, or environmental modulation, can destabilize neurodevelopmental and systemic programs, particularly during critical windows of brain maturation.

Although the MECP2 protein itself was undetectable in CSF, consistent with its nuclear localization (Zlatic et al., 2022), the observed downstream proteomic cascade supports a model of environmentally triggered *MECP2*-axis disruption as a unifying pathological driver in NS. This model gains plausibility given the broader environmental context: biotoxin exposures occurred amid prolonged conflict, displacement, malnutrition, and recurrent infections, conditions known to increase the risk of prenatal maternal immune activation (MIA). MIA, in turn, has been implicated in long-lasting epigenetic and immunological reprogramming within the offspring brain, including disruption of neurodevelopmental regulators such as MECP2 (Basil et al., 2014).

The present findings support the hypothesis that NS may represent an environmentally mediated phenocopy of *MECP2* network dysregulation, initiated prenatally by MIA and exacerbated postnatally by neurotoxic and inflammatory stressors. By integrating systems-level CSF proteomics with clinical, pathological, and ecological evidence, this study lays the groundwork for future mechanistic exploration and identifies plausible entry points for targeted therapeutic intervention.

## 2 Materials and methods

### 2.1 Study design and ethics

**Figure 1:**
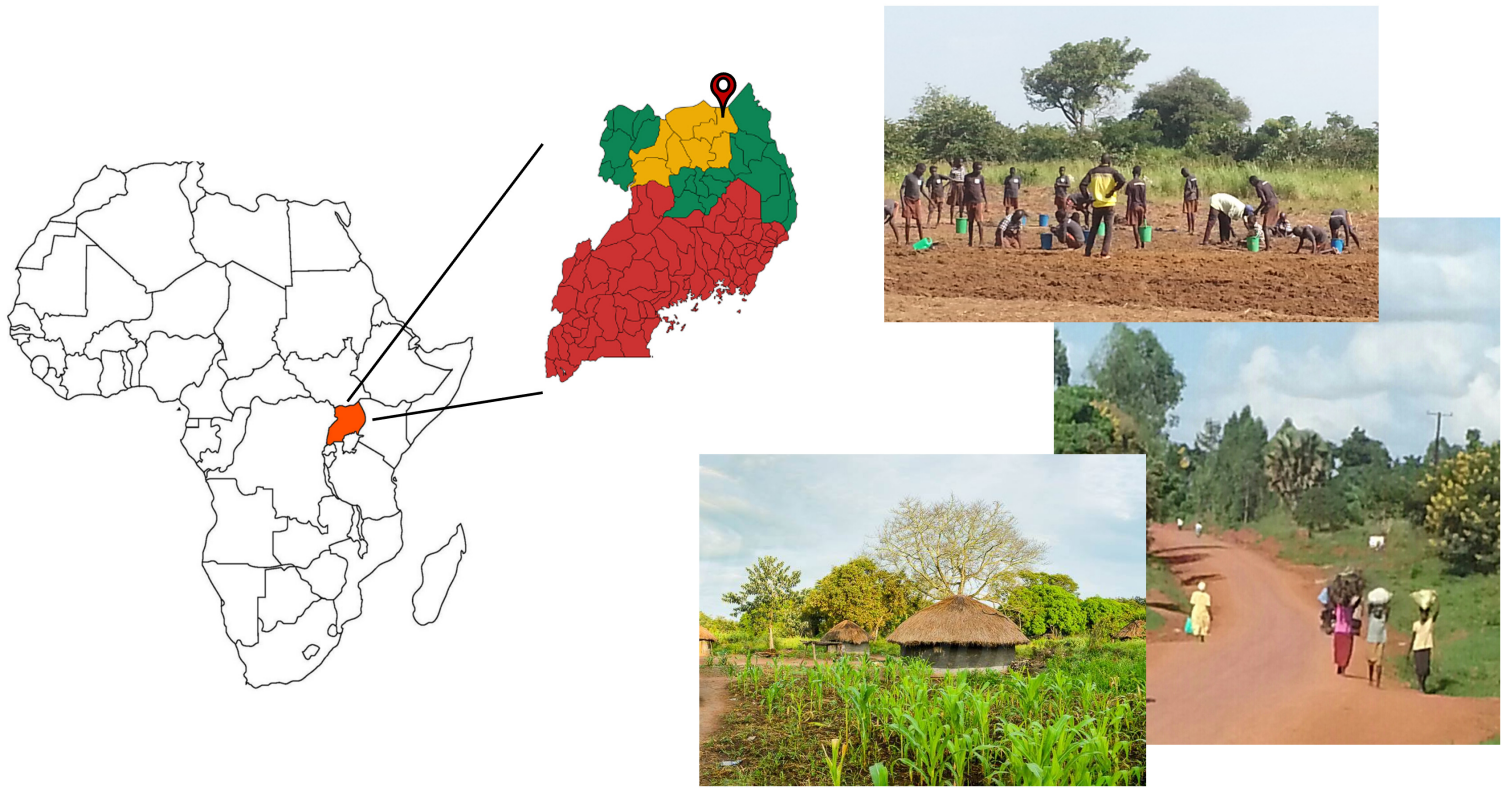
Study region and clinical site location. Map of Africa indicating Uganda in red **(left)**, with an enlarged map of Uganda **(right)** highlighting the Acholiland region (yellow) in the north (green), an area with a high burden of NS cases. The red pin marks Kitgum District, the location of the study hospital where CSF samples were collected. NS participants were recruited from Kitgum and neighboring Gulu Districts within Acholiland, as detailed in the Material and Methods Section. The figure includes a small number of photographs to provide contextual information on the environment in which participant children were born and raised.

A case-control study focused on NS was conducted among the Acholi population in the NS-prone region of Acholiland, encompassing the adjacent Gulu and Kitgum Districts of northern Uganda (Figure 1). This region is socioeconomically disadvantaged, with limited infrastructure and persistent poverty following the prolonged conflict (late 1980s-mid 2000s) between the Lord's Resistance Army and the Government of Uganda. Study participants were screened for eligibility as detailed below. Eligible individuals were temporarily admitted to Kitgum General Hospital (KGH), the region's primary referral center for epilepsy and NS, where all study procedures were carried out. Clinical assessments and CSF samples were obtained from 21 individuals diagnosed with NS (10 females, 11 males; 12–22 years; mean age 17.62 ± 3.14 years) and 10 hospital-based seizure-free Controls who underwent lumbar puncture for NS-unrelated medical concerns (2 females, 8 males; 12–28 years; mean age 20.55 ± 4.97 years). Due to the limited availability of pediatric CSF control samples, participants were included based on accessibility rather than through specific matching for age or sex. While all individuals were children or adolescents, the small sample size precluded strict demographic matching, despite efforts to optimize comparability where possible.

From this cohort, subsets of 9 confirmed NS Cases (6 females, 3 males; 12–22 years; mean age 17.7 ± 3.77 years) and 9 NS-free clinical Controls (2 females, 7 males; 12–28 years; mean age 20.1 ± 5.00) were selected for proteomic profiling (Section 2.2). Demographic and clinical characteristics are summarized in Table 1. Written informed consent was obtained from all participants or their legal guardians prior to enrollment. In the event a participant was unable to read and write, consent was obtained by fingerprint. Assent was obtained from all children and adolescents, except from those who were severely cognitively impaired. Ethical approval was granted by the School of Medicine Research and Ethics Committee (SOMREC) of Makerere University, Kampala, Uganda and the Institutional Review Board (OHSU-IRB) of the School of Medicine of Oregon Health & Science University, Portland, OR, USA. Regulatory approval was given by the Uganda National Council for Science and Technology (UNCST).

**Table 1:** Participant clinical information.

**Patient**	**Age**	**Sex**	**Medication**	**Other relevant information**
**NS Cases**				
NS-1	22	Male	NaVal, CBZ, F/A	
NS-2	12	Female	CBZ, F/A	Consistent medication adherence. Excessive appetite.
NS-4	21	Male	NaVal, F/A	Weak and dependent. Frequent head nodding.
NS-5	19	Female	CBZ, F/A	
NS-6	20	Female	CBZ, F/A	Wounds on face/forehead resulting from falls due to head nodding. Fungal infection present. Shouts or laughs prior to and sleeps after a head-nodding episode. Subsequently, she wakes up crying.
NS-7	12	Female	CBZ, F/A	Consistent medication adherence. Good appetite. Decreased seizures.
NS-8	18	Male	CBZ, F/A	Deceased
NS-9	15	Female	CBZ, F/A	Active. Helps with chores. Fair cognition
NS-11	20	Female	NaVal, CBZ, F/A	
**Controls**				
C-1	22	Male		Convulsions, unconsciousness
C-2	24	Male		Convulsions, severe malaria
C-3	12	Male		Cerebral malaria, septicemia
C-4	23	Male		Convulsions
C-5	21	Male		Severe malaria
C-7	19	Male		Meningitis
C-8	28	Female		Meningitis
C-9	14	Female		Convulsions, hysteria
C-10	18	Male		Severe malaria, meningitis

NaVal, Sodium valproate; CBZ, Carbamazepine; F/A, Folic acid.

### 2.2 Clinical information

General information and medical histories of the patients were collected, including sex, age, diagnosis, and medication history. Inclusion criteria: compliance with the diagnostic criteria for NS developed during an international 2012 conference in Kampala, Uganda (Dowell et al., 2013). Exclusion criteria: secondary seizures due to other central nervous system (CNS) disorders, such as cerebrovascular disease, traumatic brain injury, and encephalitis.

### 2.3 CSF collection and proteomic profiling

#### 2.3.1 Sample collection

Following standard aseptic methods and local anesthesia, CSF samples (2–5 mL) were collected in polypropylene tubes from the lumbar region (L3–L4), centrifuged to remove cells, and stored at −20 to −80 °C at KGH prior to their shipment on dry ice to OHSU in Portland, Oregon, USA for long-term storage at −80 °C.

#### 2.3.2 Sample preparation

CSF samples were thawed on ice, vortexed for 30 s, and 500 μL transferred into 2 mL Lobind tubes. The color of each sample was recorded, as discoloration can indicate blood contamination or other pre-analytical variation. Protein concentration was measured using a Pierce BCA assay (ThermoFisher Scientific, catalog #23225) on 10 μL of each sample, diluted to 20 μL with HPLC-grade water.

Pre-analytical validation was performed by SDS-PAGE to assess protein integrity and detect potential degradation. For SDS-PAGE analysis, 5 μg of protein per sample were dried, dissolved in 20 μL of loading buffer, and loaded onto Invitrogen NuPAGE 4%−12% Bis-Tris Mini Protein gels (Catalog # NP0335Box). Electrophoresis was conducted at 200 V constant for 50 min using BioRad SDS-PAGE standard Broad Range molecular markers (Catalog # 161-0317). Two separate 1D gels were run: one for Control samples and one for NS samples. Following overnight staining with Coomassie Brilliant Blue and destaining, gels were imaged, focusing on distinct protein bands and a prominent albumin band at 60–65 kDa, indicative of samples with good integrity.

#### 2.3.3 Depletion and concentration

CSF samples with confirmed protein integrity were subjected to high-abundance protein depletion using Agilent MARS14 spin cartridges (Catalog # 5188-6560) as per manufacturer instructions. Post-depletion, samples were buffer exchanged into 1X PBS and concentrated to a final volume of 50–100 μL using Amicon Ultra-4 centrifugal filters with a 3KDa cutoff (Catalog # UFC800324).

Protein concentration was measured pre- and post-depletion using a Pierce BCA assay (ThermoFisher Scientific, catalog #23225) and compared to non-depleted CSF to assess depletion efficiency. An average depletion efficiency of 92.1% was achieved, corresponding to an average protein recovery of 7.9% (range 3.7%−16.6%). The observed protein recovery was consistent with expected performance of the MARS14 depletion platform, which removes more than 90% of high-abundance CSF proteins to facilitate detection of lower-abundance species. Depletion procedures were standardized across all samples, and targeted high-abundance proteins were retained in downstream analyses to assess potential depletion-related bias. No evidence of systematic, group-specific distortion, or preferential protein loss was observed. Nonetheless, selective co-depletion of protein complexes or tightly associated binding partners remains a theoretical limitation of affinity-based depletion approaches.

#### 2.3.4 Proteomic analysis

##### 2.3.4.1 Protein digestion

Each sample (20 μg of protein) was digested using the ThermoFisher Scientific EasyPrep Mini-MS Sample Prep Kit (Catalog # A40006), following the manufacturer's protocol. Peptide recovery was quantified using a Pierce Quantitative Colorimetric Peptide assay (ThermoFisher Scientific, catalog #23275) to ensure consistent digestion efficiency across samples.

##### 2.3.4.2 TMT labeling and normalization

Peptides from all 18 samples were labeled with ThermoFisher TMTpro 18-plex reagents according to the manufacturer's instructions. Reporter-ion total signal per sample was determined by pooling 2 μL aliquots from each labeled sample and analyzing 2 μg of the combined peptide mixture using a Thermo Orbitrap Eclipse mass spectrometer (ThermoFisher) with a 125 min LC method. Following normalization, sample volumes were multiplexed to yield 35 μg of TMT-labeled peptides with similar total reporter-ion intensities per channel.

##### 2.3.4.3 Liquid chromatography and mass spectrometry

Standard calibration mixtures were used to confirm LC-MS system suitability. The multiplexed sample was dried and reconstituted in 40 μL of 10 mM ammonium formate (pH 9). TMT-labeled peptides were separated by 2D reverse-phase liquid chromatography/mass spectrometry (2D-LC/MS) using a Dionex NCS-3500RS UltiMate RSLCnano UPLC and Orbitrap Eclipse Tribrid mass spectrometer (Thermo Scientific).

For the first-dimension high-pH separation, peptides were eluted using sequential 20 μL injections of 17%, 20%−35%, 37%, 40%, 45%, 50%, and 90% acetonitrile (ACN) in 10 mM ammonium formate (pH 9) at a 3 μl/min flow rate, yielding 22 fractions. Eluted peptides were diluted with a mobile phase containing 0.1% formic acid at a 24 μL/min flow rate and loaded onto an Acclaim PepMap 100-micron × 2-cm NanoViper C18, 5-micron trap. After 10 min, the trap column was switched on-line to a PepMap RSLC 2-micron C18 75-micron × 25-cm EasySpray column (Thermo Scientific) for second-dimension separation. Peptides were then separated at low pH using a 7%−30% ACN gradient over 110 min in mobile phase containing 0.1% formic acid at a 300 nL/min flow rate.

Tandem mass spectrometry data were collected on an Orbitrap Eclipse Tribrid instrument (Thermo Scientific). Survey scans were performed in the Orbitrap mass analyzer, and data-dependent MS2 scans were acquired in the linear ion trap using collision-induced dissociation following quadrupole isolation. Reporter ions were detected in the Orbitrap mass analyzer using MS3 scans after synchronous precursor selection in the linear ion trap, with higher-energy collisional dissociation in the ion-routing multipole.

Complete sample processing, liquid chromatography, and mass spectrometry details are provided in Supplementary File 1. The mass spectrometry data have been deposited in the ProteomeXchange Consortium (http://proteomecentral.proteomexchange.org) via the PRIDE partner repository (Perez-Riverol et al., 2022) with the dataset identifier PXD068754.

#### 2.3.5 Nomenclature

Throughout this manuscript, official *Homo sapiens* gene symbols (non-italicized) are used as shorthand for the encoded proteins, unless the full protein name is required for clarity. The exception is MECP2, which is italicized when referring to the gene and non-italicized when referring to the protein, given its relevance to the proposed hypothesis. Corresponding gene and protein names, along with concise protein functions, are listed in Supplementary Table 1.

#### 2.3.6 Bioinformatic, pathway, and statistical analyses

The 22 binary instrument files were processed with the PAW pipeline (Wilmarth et al., 2009) (https://github.com/pwilmart/PAW_pipeline). Binary files were first converted to text format, after which Python scripts were employed to extract TMTpro reporter ion peak intensities and MS2 fragment ion spectra. Peptide identification was performed using the Comet search engine (version 2016.03) (Eng et al., 2013) with the following parameters: 1.25 Da monoisotopic peptide mass tolerance, 1.0005 Da monoisotopic fragment ion tolerance, semi tryptic cleavage allowing up to three missed cleavages, variable oxidation of methionine residues, static alkylation of cysteines, and static TMTpro modifications at peptide N-termini and lysine residues. A reference FASTA file (UP000005640; *Homo sapiens*, taxon ID 9606; canonical sequences: 20,650 proteins, one protein per gene) was downloaded from www.UniProt.org in January 2025. An additional 175 common contaminant sequences were added and sequence-reversed entries concatenated, resulting in a final protein FASTA file containing 41,650 entries. Functional annotations from www.UniProt.org were added to result tables (https://github.com/pwilmart/annotations).

Quality control was performed using Jupyter notebooks and included multiple metrics (boxplots of intensity distributions, multidimensional scaling clustering, coefficient-of-variance distributions per group, and sample-to-sample scatter plots within groups). These analyses identified sample NS5 as consistently problematic and flagged 5 additional samples with poorer QC metrics.

Relative differential abundances of protein reporter ion intensities were assessed using the Bioconductor package edgeR (Robinson et al., 2010) within Jupyter notebooks. Analyses applied Trimmed Mean of M-values (TMM) normalization, exact testing with trended variance, and Benjamini-Hochberg multiple testing corrections. Testing was performed both with NS5 alone removed and with all six low-quality samples removed. Unless stated otherwise, reported results reflect proteins consistently detected in both testing models.

While edgeR was developed for SAGE datasets, its quasi-likelihood framework and ability to model over-dispersion, make it well-suited for TMT-based proteomics, where data are derived from weighted spectral counts and exhibit semi-discrete distributions. Since the application of count-based models to continuous data requires careful normalization and statistical calibration, appropriate steps were taken to ensure robust inference.

#### 2.3.7 Protein network and functional enrichment analysis

Proteins were stratified into high-, medium-, and low-significance tiers based on adjusted *p-value* thresholds (Tier 1: *p* < 0.01; Tier 2: 0.05 > *p* > 0.01; Tier 3: 0.10 > *p* > 0.05). Each tier was analyzed independently using the online STRING database (https://cn.string-db.org/) (Szklarczyk et al., 2023) to generate protein-protein interaction (PPI) networks. Networks were constructed with a medium confidence interaction score (0.4) and evaluated for enrichment significance against random expectation. Functional enrichment was performed using Gene Ontology (GO) Biological Processes pathways, with a false discovery rate (FDR) threshold of < 0.05. STRING-derived clusters and enriched GO terms were subsequently consolidated into overarching biological themes for interpretation. Directional network analysis was additionally performed by classifying proteins as upregulated or downregulated based on fold-change, allowing integration of network connectivity with expression polarity.

## 3 Results

### 3.1 Proteomic overview of CSF in NS

Isobaric labeling-based quantitative proteomics was performed using 18-plex TMTpro labels and analyzed on an Orbitrap Eclipse Tribrid MS, yielding 456K MS2/MS3 scan cycles from the 22 LC fraction. A total of 85K peptide spectrum matches (PSMs) was identified at a 1% FDR. Those PSMs and their reporter ion intensities were mapped to 2,195 quantifiable proteins in the CSF of children with NS and of NS-free Controls; of these proteins, 544 were differentially expressed (DE) in both statistical models. The complete quantitative proteomic dataset is reported in Supplementary File 2.

Enrichment analysis of NS relative to Controls revealed NS-associated deficits in neuroimmune surveillance, proteostasis and autophagy, cytoskeletal and synaptic integrity, calcium homeostasis and mitochondrial function, mRNA processing and DNA repair, metabolic function, and neurovascular and extracellular matrix (ECM) integrity. These converging disruptions indicate a coordinated failure affecting different biological systems, within which tau pathology and neuroinflammation (not acute inflammatory activation but rather chronic neuroinflammatory imbalance) emerge as manifestations of the broader neurodevelopmental/neurodegenerative process.

### 3.2 Proteomic profiling and functional enrichment analysis

#### 3.2.1 Tiered protein network analysis

**Figure 2:**
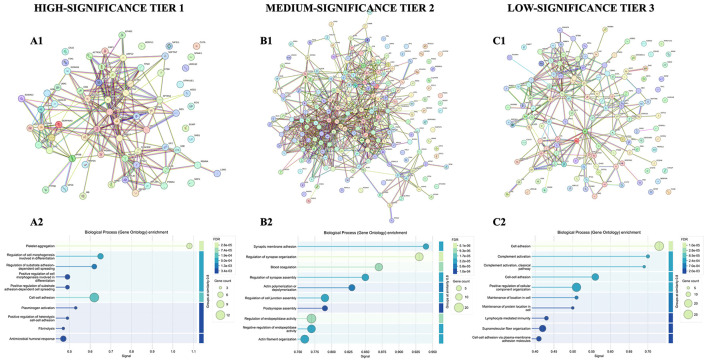
Tiered protein-protein interaction (PPI) networks and functional enrichment. **(A1–C1)** STRING PPI networks for high- (Tier 1), medium- (Tier 2), and low-significance (Tier 3) proteins. Nodes represent input proteins (with occasional STRING-added interactors), edges indicate functional associations (not limited to binding), and edge thickness reflects STRING confidence scores. Clusters denote regions of high connectivity, corresponding to functional modules. **(A2–C2)** GO Biological Process enrichment bubble plots for each tier, showing the top 10 terms ranked by FDR-adjusted p-value. Bubble size reflects the number of contributing proteins, while color indicates enrichment significance. Together, these analyses reveal a tiered architecture where Tier 1 proteins form dense coagulation-immune-cytoskeletal networks, Tier 2 extends into neuronal and stress-response modules, and Tier 3 reinforces immune-adhesion pathways at lower specificity. The differentially expressed proteins included in each tier represent the intersection of results from two independent statistical models (with 1 and 6 outliers removed, respectively), to highlight robustness across analyses.

We conducted a tiered protein-protein interaction (PPI) analysis using STRING and stratifying proteins by Benjamini-Hochberg adjusted *p-values*: Tier 1 (high significance: *p* < 0.01), Tier 2 (medium significance: 0.05 > p > 0.01), and Tier 3 (low significance: 0.10 > *p* > 0.05). Tier classification was applied to the 544 DE proteins that were statistically significant in both testing cohorts, and the FDR values were taken from the edgeR results where 6 samples were excluded. For each tier, STRING generated PPI networks, independent of expression direction. Complementary Gene Ontology (GO) Biological Processes contextualized the clusters identified, highlighting disproportionately represented biological functions. This integration allowed us to resolve both network topology and functional annotation, clarifying how dysregulated proteins interact and which biological processes they represent (Figure 2).

##### 3.2.1.1 Tier 1: high-significance proteins

The high-significance tier (106 proteins) formed a dense and highly interconnected network (69 nodes, 227 edges; average node degree 6.58, average clustering coefficient 0.489), significantly enriched beyond random expectation (*p* < 0.001). STRING identified tightly interconnected clusters, which mapped to functional nodules involved in hemostasis, cytoskeletal regulation, and innate immune defense. These included fibrinogen/thrombophilia-associated proteins, S100/annexin calcium-binding proteins, antimicrobial metal-sequestering proteins, and actin cytoskeleton regulators such as Rho GTPases and the Arp2/3 (ACTR2/3) complex (Figure 2 A1). GO enrichment confirmed these themes, highlighting platelet aggregation, fibrinolysis, cell-cell adhesion, and apoptotic regulation (Figure 2 A2). Thus, Tier 1 captures a tightly interwoven set of pathways governing coagulation, cytoskeletal remodeling, and host defense, key processes in disease pathology.

##### 3.2.1.2 Tier 2: medium-significance proteins

The medium-significance tier (260 proteins) also produced a significantly enriched network (195 nodes, 766 edges; average node degree 7.86, average clustering coefficient 0.409, *p* < 0.001). STRING revealed distinct clusters, which aligned with functional modules involved in synaptic organization (e.g., neurexins, neuroligins, postsynaptic complexes), cytoskeletal remodeling (e.g., Rho GTPases, Arp 2/3 complex), protein quality control (e.g., proteasome, chaperones), and immune-coagulation processes (Figure 2 B1). GO enrichment reinforced these associations, highlighting synaptic signaling, trans-synaptic membrane adhesion, regulation of protein metabolism, coagulation, complement activation, proteolysis, and apoptosis (Figure 2 B2). Collectively, Tier 2 proteins extend the Tier 1 pathways, suggesting that neuronal connectivity, cytoskeletal dynamics, and protein homeostasis represent additional layers of vulnerability linked to systemic immune and coagulation networks.

##### 3.2.1.3 Tier 3: low-significance proteins

The low-significance tier (178 proteins) yielded a less compact but still enriched network (131 nodes, 261 edges; average node degree 3.98, average clustering coefficient 0.405, *p* < 0.001). STRING revealed smaller clusters, which mapped to functional modules associated with complement and coagulation cascades, opsonization, and protein-lipid complex assembly (Figure 2 C1). GO enrichment emphasized cell adhesion (including cell-cell adhesion and adhesion mediated by plasma membrane molecules), complement activation (including the classical pathway), immune effector responses, stress responses, proteolysis regulation, cell morphogenesis, and neuronal survival (Figure 2 C2). Although these associations are less specific than those in higher tiers, they appear to recapitulate the recurring biological architecture with weaker associations converging on cell adhesion, immune-coagulation homeostasis, and stress regulation.

##### 3.2.1.4 Integrated summary across tiers

Together, these tiered analyses delineate a hierarchical biological architecture underlying the NS proteome. The high-significance proteins (Tier 1) identified foundational disruptions in coagulation, immune defense, and cytoskeletal integrity. Medium-significance proteins (Tier 2) extended this molecular signature into the domains of neuronal connectivity and protein quality control, processes that functionally depend on the systemic stability established in Tier 1. Finally, the lower-significance proteins (Tier 3), although statistically more diffuse, consistently reiterated these same biological modules, particularly cell adhesion, immune-coagulation, and stress responses. This recurrence across independent statistical tiers distinguishes these findings from stochastic noise; the fact that the same functional themes persist regardless of stringency suggests they represent central, interconnected nodes of vulnerability within the disease network. Collectively, this tiered architecture reflects a systemic collapse of homeostatic regulation.

##### 3.2.1.5 Directional network analysis

To further refine this architecture, proteins were classified by expression polarity, distinguishing those significantly increased in NS from those depleted relative to Controls. Directional mapping provided a functional overlay to the tiered structure, revealing that the most interconnected network nodes identified in earlier tiers were not only central but also directionally suppressed in NS CSF. Core homeostatic systems (e.g., complement cascade, proteasomal subunits, autophagic machinery, cytoskeletal components, and mitochondrial regulators) exhibited broad, coordinated downregulation. This systemic repression underscores a widespread failure of processes essential for cellular stability, bioenergetics, and survival. In contrast, a smaller, distinct set of selectively upregulated proteins emerged. These included factors associated with stress adaptation and vascular remodeling (e.g., VEGF-C) and mediators of synaptic and neurodevelopmental plasticity (e.g., NLGN1, ASTN2), mapping largely into Tier 2 functional modules.

This directional analysis integrates with the tiered framework to define a bipartite signature of dysregulation: a profound, network-wide suppression of fundamental homeostatic systems at the core, potentially countered by a marginal, localized induction of proteins involved in neuroplasticity and stress response.

#### 3.2.2 Biological process enrichment analysis (GO) of differentially expressed proteins

To characterize the physiological implications of the NS proteome, we performed Gene Ontology (GO) enrichment analysis on all differentially expressed proteins. While the network analyses in Section 3.2.1.5 focused on the hierarchical and directional relationships between individual nodes, this functional profiling identifies the collective biological pathways and cellular systems most consistently divergent in NS. By grouping proteins into higher-order functional categories, this analysis reveals how multiple, discrete molecular imbalances converge into systemic vulnerabilities, providing a comprehensive view of the multisystem failure defining the NS proteomic signature.

##### 3.2.2.1 Immune silencing and tolerogenic skewing

NS samples exhibited a broad-spectrum depletion of immune regulators spanning both innate and adaptive compartments. Levels of acute-phase proteins (e.g., truncated SAA1, SAA2, ITIH4, SERPINA3, ORM1/2) as well as the macrophage activation marker sCD163 were significantly reduced in NS relative to Control CSF (Sánchez-Navarro et al., 2021; Plevriti et al., 2024; Papareddy and Herwald, 2025; Luo et al., 2015). The diminished abundance of these typically plasma-derived proteins is consistent with reduced CNS entry or systemic attenuation of the acute-phase response within the neurovascular niche. This pattern was accompanied by a widespread downregulation of the complement cascade (e.g., C2, C4A, C5, C6, C8/C8A/B, CFB/Bb fragment, CFI, C4BPA/B, CFP, FCN3) (Lee et al., 2019), indicating suppression of both classical and lectin complement pathways.

Additional reductions were observed in chemotactic and recruitment-related factors (e.g., S100A8/9, LBP, SAA, PF4, PPIA) together with cell adhesion proteins (AMICA1, ITGB2) (Sprenkeler et al., 2022; Su et al., 1995; Soriano et al., 2020; Ngo et al., 2023; Dong et al., 2025; Bouti et al., 2021; Nigro et al., 2013), consistent with impaired leukocyte trafficking and cellular surveillance. Within the adaptive compartment, declines were noted in key regulators such as IGHM and the inhibitory phosphatase PTPN6 (Jones et al., 2020; Kiratikanon et al., 2022), as well as proteins governing immune-related transcriptional and post-transcriptional control, such as SET and the RNA helicase DDX39B (Xu et al., 2018).

A central theme within this profile was the concurrent downregulation of pro-inflammatory signaling. This was evidenced by depletion of the inflammasome adaptor PYCARD (Johnson et al., 2023) and a coordinated reduction in NF-κB pathway components and their responsive factors, including alarmins (e.g., S100-A8/A9) and the antioxidant enzyme HMOX1 (Yu et al., 2020; Zhang et al., 2017; Bonizzi and Karin, 2004; Wang et al., 2018; Paine et al., 2010). This immune attenuation coincided with loss of core proteostatic executors, namely the AAA^+^ ATPase VCP, a mediator of protein extraction and degradation (Meyer et al., 2012), and the constitutive chaperone HSPA8, both essential for the cellular response to proteotoxic stress. The simultaneous collapse of proteostatic and inflammatory pathways points to a brain that has lost its capacity to sense or respond to aggregate-induced damage.

Linked to this homeostatic failure was depletion of the immunophilin FKBP1A. Beyond its peptidyl-prolyl isomerase activity in protein folding, FKBP1A modulates mTOR signaling (Saxton and Sabatini, 2017); its reduction, coupled with downregulation of other core mTOR pathway components (Section 4.2), identifies a defect in the metabolic and translational machinery associated with neuroimmune activation. Further characterizing this profile was downregulation of the cellular stress marker NPM1 and the immunoproteasome-specific subunit PSME1 (Schwarz et al., 2000). Depletion of PSME1 is consistent with impaired immunoproteasome function and reduced capacity for canonical MHC class I antigen processing.

In contrast, a selective subset of immunomodulatory and tolerogenic proteins was elevated in NS vs. Control CSF. These included SFTPD, a collectin that modulates innate signaling to limit pro-inflammatory cytokine production (Wright, 1997; Shamim et al., 2024), and FCER2, a low-affinity IgE receptor that contributes to negative feedback regulation and the maintenance of immune homeostasis and B cell tolerance (Rosenwasser and Meng, 2005; Engeroff and Vogel, 2021). Notably, although many components of the antigen presentation machinery were reduced, HLA-C, a classical MHC class I molecule, was elevated. This profile was further marked by increased levels of the inhibitory checkpoint CD200, the neuroimmunomodulator PRL (Jayakumar et al., 2022), and multiple non-canonical signaling factors associated with tissue remodeling and developmental signaling, including C1QTNF4, ROR1 (Paganoni et al., 2010; Chanda et al., 2021), and the synaptogenic glypican GPC6 (Filmus, 2022).

Taken together, these findings define a proteomic landscape of global immune and stress-response suppression alongside selective induction of immunoregulatory checkpoints and developmental signals. This pattern delineates a “hushed” neuroimmune environment, in which systemic immune cues are muted or excluded, consistent with a pathological shift toward a tolerogenic and developmentally immature state in the NS brain.

##### 3.2.2.2 Proteostatic debt and autophagic collapse

Both major intracellular clearance pathways, the ubiquitin-proteasome system (UPS) and the autophagy-lysosome pathway, exhibited significant downregulation in NS relative to Control CSF. Core UPS components, including the E1 activating enzyme UBA1, the E2 conjugating enzyme UBE2K, and the deubiquitinase UCHL1, were reduced alongside multiple proteasomal subunits. These findings identify a systematic failure of the machinery required for ubiquitin-tagging and proteasomal degradation (Hershko and Ciechanover, 1998; Finley, 2009).

Concurrently, the autophagy-lysosome axis showed a coordinated depletion of proteins previously identified as foundational for proteostatic support, including HSPA8 and VCP, alongside HMGB1, and PARK7. These factors are essential for autophagosome formation, maturation, and lysosomal fusion (Mizushima et al., 2008; Nixon, 2013). The loss of VCP, a mechanochemical segregase that links the UPS and autophagic pathways (Chu et al., 2023; Ebrahimi-Fakhari et al., 2012), further underscores the extent of protein clearance failure across both systems.

Additionally, reduced levels of several vacuolar H^+^-ATPase subunits (ATP6V1A/B2/E1) were observed. These subunits are critical for maintaining lysosomal acidification, a process fundamental to autophagic flux, proteostasis, and immune regulation (Collins and Forgac, 2020).

Together, these findings define a coordinated downregulation of proteostasis-associated mechanisms in NS. This profile indicates a reduced capacity for the intracellular clearance of misfolded proteins and cellular debris, reinforcing the state of cellular stress and signaling failure previously observed.

##### 3.2.2.3 Proteostasis-linked cytoskeletal destabilization

Over 40 cytoskeleton-associated proteins were reduced in NS relative to Control CSF, with the largest representation involving proteins governing microtubule and actin filament architecture. Notably, microtubule-associated protein 2 (MAP2), a dendrite-enriched microtubule stabilizer (Harada et al., 2002); α-synuclein (SNCA), a synaptic protein that modulates cytoskeletal assembly and dynamics (Carnwath et al., 2018; Seebauer et al., 2022; Amadeo et al., 2021); and TUBA1A, an α-tubulin isoform integral to microtubule structure (Janke and Magiera, 2020; Kreis, 1987), were all significantly downregulated. The concurrent depletion of these stabilizers and other core structural subunits identifies a substantial compromise of microtubule integrity and, by extension, axonal/dendritic transport capacity.

Cytoskeletal instability also extended to the actin network, where multiple regulators of actin polymerization (e.g., PFN1, CFL1, ACTR2/3, coronins, TWF2) were markedly reduced. In parallel, anchoring and scaffolding proteins critical for mechanical stability and spatial organization (e.g., FLNA, TLN1, spectrins, VCL, non-conventional myosins, VASP), were similarly diminished. These collective changes characterize a broad perturbation of the excitatory synaptic architecture, consistent with altered dendritic spine dynamics (Bai et al., 2023; Dent et al., 2011; van der Kooij et al., 2016; Sheng and Pak, 1999).

Together, these alterations define a global structural attrition of cellular scaffolds in NS. This collapse occurs alongside the widespread impairment of protein quality control machinery described in Section 3.2.2.2, delineating a proteomic signature in which loss of homeostatic maintenance is tightly coupled to erosion of the physical neuropil. While CSF proteomics cannot definitively assign cell-type origin or directionality of change, the coordinated reduction of these conserved structural proteins, spanning both microtubule and actin compartments, suggests that the proteostatic burden in NS is associated with systemic compromise of architectural integrity in both neurons and supporting glial cells.

##### 3.2.2.4 Calcium overload, mitochondrial failure, and endosomal attrition

NS samples showed evidence of profound disruption in calcium regulation, mitochondrial integrity, and endocytic trafficking relative to Control CSF. Proteins involved in membrane-associated calcium transport were elevated in NS CSF, including SARAF, a modulator of store-operated calcium entry (SOCE) (Kodakandla et al., 2023; Dagan and Palty, 2021), and SLC8A1, the principal sodium/calcium exchanger responsible for bidirectional extrusion of cytosolic calcium (Lytton, 2007). The selective upregulation of SARAF and SLC8A1 identifies a reactive homeostatic effort to mitigate cytosolic calcium overload through enhanced membrane-mediated extrusion. In contrast, multiple intracellular calcium buffers and sensors, including calbindin (CALB1), calmodulin (CALM1), and TPT1 were reduced (Kim et al., 2000; Bommer, 2017; Xia and Storm, 2005; Schwaller, 2010). This divergence is consistent with a state of chronic calcium stress, where the exhaustion of internal sequestration capacity triggers a compensatory, yet ultimately inefficient, reliance on extrusion machinery.

Concomitantly, evidence indicative of mitochondrial dysfunction was observed, including reduced levels of oxidative phosphorylation-associated proteins (COX6B1, ECI1) and key antioxidant enzymes such as catalase (CAT) and peroxiredoxin (PRDX). Levels of PARK7, a mitochondrial stress-response factor that supports Complex I stability and reactive oxygen species (ROS) detoxification (Zhang et al., 2016, 2021), were also decreased, consistent with compromised mitochondrial resilience.

Linked to these metabolic deficits, several regulators of endosomal dynamics and Rab GTPase cycling were significantly downregulated in NS vs. Control CSF. These included the early endosome-associated RAB5C, the recycling endosome regulator RAB11A (Stenmark, 2009), and the Rab GDP-dissociation inhibitor RABGDIB, which is essential for the functional recycling of the Rab network (Cherfils and Zeghouf, 2013). The downregulation of these factors, in the context of impaired autophagic flux (Section 3.2.2.2, Ao et al., 2014), indicates a deep disruption of the endocytic-lysosomal axis.

Together, these findings outline a profile of dysregulated calcium homeostasis, mitochondrial functional impairment, and endosomal attrition in NS, suggestive of intertwined disturbances in calcium handling, energy production, and intracellular trafficking.

##### 3.2.2.5 Maladaptive inhibitory shift and network rigidity

Proteomic profiles in NS relative to Control CSF revealed an increased abundance of GABBR1, the ligand-binding subunit of the heterodimeric GABA-B receptor that mediates prolonged inhibitory neurotransmission via G-protein-coupled signaling (Chalifoux and Carter, 2011). This upregulation was accompanied by elevated levels of synaptic organizers and ECM components, including the trans-synaptic adhesion molecules NLGN1/3, the matrix-organizing RELN, and TNR, a major component of the perineuronal ECM scaffold. Additionally, the axon guidance protein SEMA6A, a member of the semaphoring family, was significantly elevated.

Collectively, these results identify a selective enrichment of proteins governing inhibitory signaling and synaptic adhesion in NS. The concurrent increase in these factors defines a proteomic signature with elevated inhibitory capacity and enhanced cell-matrix and trans-synaptic junctional representation.

##### 3.2.2.6 Tau cleavage and the phosphorylation-aggregation loop

Altered representation of pathways associated with amyloid precursor protein (APP) proteolysis and tau regulation was observed in NS relative to Control CSF. Elevated levels of the neurotoxic APP C31 fragment, a cleavage product attributed to caspase 3 (CASP3) activity, were detected in NS, identifying dysregulated APP processing (Tawa et al., 2004). In experimental models, this fragment is a potent inhibitor of protein phosphatase 2A (PP2A), a primary phosphatase responsible for tau dephosphorylation. Therefore, its elevation in NS is consistent with a biochemical environment conducive to tau hyperphosphorylation (Park et al., 2012).

Linking back to the calcium overload observed in Section 3.2.2.4, upregulation of CAPN11, a calcium-dependent protease implicated in tau truncation (Descalzi et al., 2017; Ferreira and Bigio, 2011), was also observed (model excluding the NS5 outlier). In parallel, proteins involved in microtubule stability and proteostasis, including MAP2, the adapter YWHAQ, and the chaperone HSP90AA1, were significantly reduced. Additional declines in nuclear transport-associated regulators (e.g., RBBP4, TPR) (Frosst et al., 2002; Aksenova et al., 2020) are consistent with recent evidence linking nuclear pore complex dysfunction to tau-mediated neurotoxicity (Eftekharzadeh et al., 2018).

Collectively, these findings identify a coordinated disruption of tau-related regulatory pathways in NS. However, total tau concentrations remained comparable between NS and Control groups, indicating that changes in tau processing and modification may not translate into shifts in overall CSF tau abundance in this dataset. Phosphorylated and truncated tau isoforms were not detected, which may reflect either a true biological absence or, more likely, the analytical limitations of the mass spectrometry (MS) workflow. Without targeted enrichment or specialized phospho-proteomic pipelines, low-abundance, labile, or post-translationally modified tau species are often below detection thresholds in discovery proteomics (Mayya and Han, 2009; Ptacek and Snyder, 2006). Of note, the concurrent downregulation of proteostatic clearance executors (Section 3.2.2.2) and cytoskeletal stabilizers (Section 3.2.2.3) suggests a scenario in which altered tau conformers could be retained within the brain parenchyma rather than freely diffusing into the CSF compartment.

##### 3.2.2.7 Transcriptional silencing and DNA repair inefficiency

Broad alterations across the gene-expression cascade were also observed in NS vs. Control CSF. Downregulated proteins included transcription-associated factors (e.g., MNDA, HDGF, HMGA1, ILF2, TCEAL6) (Zhou et al., 2004; Yie et al., 1999; Wei et al., 2021) and a large group of RNA-processing regulators. The latter group comprised multiple hnRNPs, snRNPs, and the splicing factors SRSF2 and PTBP1 (Li et al., 2023; Fisette et al., 2010), as well as mediators of the TREX mRNA export complex, such as DDX39B and SRSF1 (Li et al., 2023; Hirano et al., 2023). Concurrently, several translational machinery components, including the elongation factors EIF5A1 and EEF2, and several tRNA ligases, were significantly reduced.

In contrast, upregulation of a subset of stress-responsive and glial-associated proteins was observed, including the ER co-chaperone SIL1 (Labisch et al., 2018), the glial modulator AEBP1 (Shijo et al., 2018), and the neurovascular mediator VEGF-C (Ju et al., 2019; Lange et al., 2016).

Evidence of metabolic redirection was also apparent, with reduced abundance of enzymes involved in *de novo* purine biosynthesis (PAICS) (Williamson et al., 2017) and the purine salvage pathway (PNP) (Tsui et al., 2022). The additional reduction of S-adenosylhomocysteine hydrolase (AHCY), an enzyme critical for maintaining the cellular methylation potential, points to a biochemical environment prone to epigenetic dysregulation (Pavičić et al., 2023). This reduction in nucleotide biosynthetic capacity, coupled with depletion of AHCY, indicates a limitation in nucleotide availability for RNA transcription and DNA repair, alongside a diminished capacity for epigenetic methylation.

Finally, several DNA-repair and genomic surveillance proteins, including UBE2N, the double-strand break sensor XRCC5 (Cai et al., 2023), the apurinic/apyrimidinic endonuclease APEX1 (Pei et al., 2019; Yuan et al., 2025), and the single-stranded DNA-binding protein RPA1 (Kim et al., 1996), were decreased in NS relative to Control CSF.

Collectively, these CSF proteomic profiles are consistent with a widespread repression of transcriptional, splicing, translational, and DNA-maintenance pathways in NS. This coordinated downregulation delineates a molecular landscape indicative of reduced biosynthetic capacity and compromised genomic maintenance.

##### 3.2.2.8 Lipid metabolic drift and the collapse of vesicular acidification

Widespread alterations in lipid metabolism and ionic homeostasis were observed in NS relative to Control CSF. Several enzymes linked to fatty acid β-oxidation (Houten and Wanders, 2010), including ECI1 (mitochondrial) and ACOX1 (peroxisomal) (Chung et al., 2020) were downregulated, consistent with reduced capacity for fatty acid catabolism. Detoxifying enzymes involved in clearing reactive lipid peroxidation products, such as AKR1A1/B1 and ALDH1A1 (Penning, 2015; Ahmed Laskar and Younus, 2019; Karan et al., 2023), were also reduced. Components of lipid signaling pathways, including leukotriene (LTA4H) (Adams et al., 2023) and prostaglandin (PTGES3) enzymes, were diminished, suggesting altered eicosanoid metabolism.

By contrast, proteins associated with sphingolipid turnover and lysosomal lipid trafficking (e.g., ASAH1, GALC, NPC2) (Feo et al., 2025; Xicoy et al., 2019; Di Pardo and Maglione, 2018; Walkley and Suzuki, 2004), along with carboxyl ester lipase (CEL), a broad-specificity lipid hydrolase involved in extracellular lipid processing, were elevated. Importantly, ACLY, a key cytosolic enzyme that links carbohydrate metabolism to *de novo* lipid precursor synthesis (Biju et al., 2024), was significantly reduced, consistent with an impairment of lipid metabolic flux.

Ion transport and redox balance were likewise affected. Multiple vacuolar-type H^+^-ATPase subunits (ATP6V1A/B2/E1), essential for vesicular acidification and endo-lysosomal function, were reduced in NS vs. Control CSF (Morel and Poëa-Guyon, 2015). In parallel, antioxidant enzymes such as catalase (CAT) (Anwar et al., 2024) and the myeloid-derived inflammatory enzyme myeloperoxidase (MPO) (Chen et al., 2020; Yu et al., 2016) alongside CLIC1, a stress-responsive chloride channel involved in maintaining ionic and redox balance (Gururaja Rao et al., 2020), were all downregulated. The depletion of MPO points to a broad suppression of myeloid-cell activation and immune infiltration, while the reduction in ATP6V subunits identifies a critical failure in vesicular acidification.

Together, these proteomic patterns indicate a failure of lipid biosynthetic and detoxifying pathways, accompanied by elevated sphingolipid turnover and compromised organelle acidification. This combination is indicative of progressive membrane destabilization and lysosomal exhaustion in NS.

##### 3.2.2.9 Carbohydrate metabolism failure and the loss of redox defense

Multiple key enzymes involved in glycolysis and gluconeogenesis (e.g., ENO1, PKM, PGAM1, ALDOB/C, PGK1, GPI) were downregulated in NS relative to Control CSF. This suppression extended to PYGL, the glycogen phosphorylase responsible for glycogen mobilization, identifying a broad disruption in carbohydrate metabolic and storage pathways (Johnson et al., 2020; Ryu et al., 2021).

Enzymes central to NADPH biosynthesis were also diminished, including PGD, a pivotal catalyst within the oxidative branch of the pentose phosphate pathway (PPP), and IDH1, a primary cytosolic generator of neural NADPH (Biedermann et al., 2019). The reduction of TALDO1, a core component of the PPP's non-oxidative branch involved in recycling glycolytic intermediates (Qian et al., 2008), further characterizes a systemic PPP impairment.

Collectively, these proteomic signatures define a coordinated downregulation across glycolytic, gluconeogenic, glycogenolytic, and NADPH-generating circuits in NS, consistent with an emergent energetic deficit and a compromised capacity to maintain a reducing environment. Such a profile points to a diminished homeostatic reserve to sustain redox recycling, potentially exacerbating the cellular response to the oxidative and proteotoxic stressors identified in previous sections.

##### 3.2.2.10 Neurovascular fragility and extracellular matrix (ECM) disarray

Comprehensive downregulation of proteins integral to vascular stability and ECM homeostasis was observed in NS relative to Control CSF. Core constituents of the coagulation and fibrinolytic cascades, including fibrinogen chains (e.g., FGA, FGB, FGG), plasminogen (PLG), and kallikrein-related peptidases (e.g., KLKB1), together with annexin A5 (ANXA5), were markedly reduced (Erickson et al., 2024; Ledesma et al., 2000; Kidana et al., 2018; Bartolome et al., 2020). The depletion of these typically plasma-derived proteins is consistent with perturbations in vascular supply and altered BBB dynamics. Simultaneous suppression of matrix metalloproteinases (MMP3/8/9), critical for ECM remodeling (Stamenkovic, 2003), together with hemostatic factors central to vascular integrity (e.g., FGG, F5, F9, F11, F13A1) (De Luca et al., 2017; Fisher, 2013), further indicates compromised ECM remodeling and hemostatic function.

In contrast, proteins associated with stress-induced vascular remodeling, including the angiogenic mediator VEGF-C and the pro-inflammatory cytokine TNFSF12 (Khan et al., 2025), were upregulated, consistent with activation of angiogenic and inflammatory signaling.

Collectively, these proteomic profiles characterize a destabilization of neurovascular architecture in NS. The concurrent downregulation of MMPs aligns with a blunted capacity for regulated proteolytic remodeling, suggesting a shift in ECM dynamics toward pathological attrition. These findings are indicative of neurovascular compromise and engagement of reactive, pro-inflammatory vascular remodeling programs in NS.

## 4 Discussion

Integrated CSF protein profiling has been instrumental in defining molecular signatures across tau-related disorders, particularly Alzheimer's disease (AD) (Blennow et al., 2010; Higginbotham et al., 2020; Del Campo et al., 2022; Horie et al., 2023; Wang et al., 2025). To our knowledge, this is the first such study comparing individuals with and without NS. While preliminary, these findings challenge the prevailing view of NS as a primarily idiopathic epileptic encephalopathy. Instead, they suggest that NS involves a systemic architectural disruption, with proteomic and clinical signatures overlapping those observed in *MECP2*-related conditions (Section 4.2 and Supplementary Table 2).

Notably, MECP2 is increasingly recognized for its integrative role in immune tolerance, transcriptional regulation, and synaptic homeostasis. In AD and related tauopathies, even modest shifts in MECP2 activity have been linked to disease onset and progression (Maphis et al., 2017; Kim et al., 2019). Reinforcing this, animal models demonstrate that *MECP2* triplication increases tau expression, phosphorylation, and neurodegeneration (Vilvarajan et al., 2023), whereas, in neuronal cultures, *MECP2* knockdown reduces both total and phosphorylated tau (Maphis et al., 2017). Together, these data position MECP2 as a plausible upstream node in NS and a potential point of therapeutic entry within the broader tauopathy continuum.

This hypothesis is supported by the proteomic architecture observed in our dataset. Although many of the 544 DE proteins exhibited modest fold changes, the consistency of these patterns across statistical tiers reflects a distributed perturbation profile characteristic of complex neurodegenerative conditions. In such models, the cumulative impact of low-amplitude deviations across interdependent metabolic and structural systems, rather than isolated high-magnitude failures, underlie pathological progression.

Critically, NS diverges from age-associated disorders, such as AD, in its temporal dynamics. In NS, molecular disruption does not unfold over decades of attrition but within a compressed window of developmental vulnerability. Within this selective timeframe, convergent environmental insults likely amplify the impact of otherwise subtle proteomic shifts, precipitating a rapid transition from homeostatic fragility to irreversible clinical regression.

### 4.1 Reframing the pathogenesis of NS: a hypothesis for MIA-*MECP2* axis dysregulation

While genetic screening for *MECP2* mutations or duplications has not yet been performed in NS-affected populations, the geographical clustering, outbreak dynamics, and familial co-occurrence observed in NS suggest that shared environmental exposures, rather than strictly heritable factors, are central to disease etiology. These exposures are unlikely to be singular or uniform: while biotoxins (e.g., mycotoxins, cyanotoxins) remain strong environmental candidate triggers of NS, additional stressors, including nutritional deficits, recurrent infections, and socio-ecological adversity, must also be considered.

Within this ecological backdrop, maternal immune activation (MIA) emerges as a biologically plausible upstream mediator, capable of integrating the diverse environmental stressors associated with NS. MIA can induce persistent immune and epigenetic reprogramming during sensitive windows of fetal neurodevelopment. Notably, MIA is known to promote immune-tolerance phenotypes and may precipitate *MECP2* dysregulation via inflammation-driven epigenetic remodeling (Basil et al., 2014). However, *MECP2* itself, particularly when overexpressed, can also exert direct immunosuppressive effects, as demonstrated in MDS (Section 4.2), thereby raising a fundamental question: Does MIA independently give rise to both immune tolerance and *MECP2* dysregulation as parallel outcomes, or is *MECP2* instability itself the primary inflection point from which immune silencing and synaptic collapse cascade?

Based on our findings, we propose a convergent trajectory in which MIA primes the pathogenic landscape while *MECP2* dysregulation amplifies and entrains these early immune-epigenetic disruptions into a self-reinforcing degenerative cascade. Within this framework, NS does not conform to classical models of infectious or autoimmune disease. Rather, it reflects a non-genetic, environmentally primed disruption of a multi-axial system where immune-epigenetic memory and *MECP2* regulatory failure converge.

### 4.2 Cross-domain parallels between NS and *MECP2*-duplication syndrome (MDS)

Striking parallels exist between NS and MDS across clinical, molecular, immunological, electrophysiological, and neuropathological domains (Supplementary Table 2). Both syndromes feature atonic head-nodding seizures, hypotonia, growth failure, feeding difficulties, delayed puberty, psychomotor regression, intellectual disability, tauopathy, recurrent respiratory infections, cold extremities, shortened lifespan, and pronounced autism spectrum traits (Spencer et al., 2024; Ramocki et al., 2009; Arony et al., 2018a; Kitara et al., 2019).

Food- or cold-induced head drops, pathognomonic in NS (Spencer et al., 2024), mirror the axial atonia and eating-triggered head drops reported in MDS (Lorenzo et al., 2021; Li et al., 2017; de Palma et al., 2012), personal communication with Dr. Dick Sobsey, John Dosseter Health Ethics Centre, University of Alberta, Canada). These overlapping features likely originate from frontal-central cortical disruption (Lorenzo et al., 2021; Vercellino et al., 2021), consistent with the motor deficits and cortical laminar disorganization documented in NS (Pollanen et al., 2018; Colebunders et al., 2023).

Shared electroencephalographic abnormalities [slow or sharp-and-slow waves, electrodecrement, and ictal gamma bursts in NS (Mazumder et al., 2022); multifocal discharges and background slowing in MDS (Vignoli et al., 2012)], indicate not merely cortical hyperexcitability but systemic network desynchronization within structurally compromised circuits (Deng et al., 2024). This interpretation is supported by animal models: both *MECP2*-under and -overexpressing mice display hippocampal hypersynchrony preceding seizures (Lu et al., 2016), while *MECP2*-overexpressing primates exhibit reduced β-band coupling across fronto-parieto-occipital networks, correlating with motor dysfunction (Cai et al., 2020). Interestingly, similar large-scale connectivity disruptions are a hallmark of autism spectrum disorders (ASD) (Urbain et al., 2016), which share several features with NS, including developmental regression, seizures, motor dysfunction, and impaired social and cognitive integration (Arony et al., 2018a; Kitara et al., 2019).

Molecularly, NS and MDS display overlapping cytoskeletal, vesicular, and immune vulnerabilities, with dysregulated Wnt and NF-κB signaling emerging as prominent signatures (Pascual-Alonso et al., 2024). In NS relative to Control CSF, upregulation of the non-canonical Wnt receptor ROR1 (Endo et al., 2022) alongside ECM stabilizers RELN and TNR (Maguire, 2018; Pintér and Alpár, 2022; Soles et al., 2023), suggests enhanced crosstalk between non-canonical Wnt pathways and the ECM. Importantly, WNT5A → ROR1 signaling has been shown to engage CaMKII, triggering calcium influx (Chen et al., 2018). In the context of the SARAF/SLC8A1-mediated reactive calcium homeostasis (Section 3.2.2.4, Section 4.5.3), this pattern raises the possibility that non-canonical Wnt signaling contributes to, rather than relieves, calcium-mediated stress. Mechanistically, ROR1 upregulation may represent a compensatory structural response to cytoskeletal and ECM perturbations, aimed at preserving neuropil integrity. However, the metabolic and ionic cost of sustained non-canonical Wnt engagement, particularly its link to CaMKII and calcium influx, may inadvertently prolong or amplify calcium dyshomeostasis. In contrast, downregulation of YWHAQ alongside elevated levels of Wnt antagonists (e.g., WIF1, KREMEN1) indicates suppression of canonical Wnt/β-catenin signaling, a pathway essential for synaptic patterning and plasticity (Hu et al., 2008; Mao et al., 2002). Together, these observations are consistent with a model in which NS engages a shift away from canonical Wnt/β-catenin activity toward non-canonical Wnt signaling, perhaps as a developmental or structural buffer. This trade-off may transiently support aspects of structural resilience at the cost of exacerbating maladaptive ionic and excitability profiles within neural networks.

The NF-κB immune axis provides a second point of convergence (Pascual-Alonso et al., 2024). In Rett syndrome, NF-κB is aberrantly activated via IRAK1, a kinase central to innate immunity (Kim et al., 2024). This leads to impaired dendritic growth, a phenotype reversible by pathway suppression (Kishi et al., 2016). In contrast, MDS shows recurrent infections and blunted Th1 responses without overt NF-κB hyperactivation, even when *IRAK1* is co-duplicated (Gottschalk et al., 2023; Rizvi et al., 2024), indicating a hyporesponsive NF-κB state. This pattern is mirrored in NS CSF, with downregulation of a broad spectrum of immune and stress-responsive proteins (e.g., S100A8/A9, HMOX1, PYCARD, VCP, proteasomal subunits, HSPA8, FKBP1A, NPM1) alongside increased tolerogenic and developmental mediators (e.g., CD200, PRL, C1QTNF4, ROR1, GPC6). Notably, reduced levels of S100A8/A9 (alarmin/DAMP signaling) and PYCARD (inflammasome adapter assembly) suggest a failure to prime and execute canonical NF-κB/NLRP3 inflammatory responses. Coupled with depletion of HMOX1 and proteasomal components, these findings align with a profile of active neuroimmune suppression and diminished pro-inflammatory signaling in NS, rather than classical immune exhaustion.

Together, NS and MDS exhibit parallel dysregulation of Wnt-calcium and NF-κB-immune pathways, not as isolated defects but as interdependent network failures. These upstream perturbations likely converge on shared downstream outcomes, including proteotoxic overload, cytoskeletal breakdown, oxidative stress, and synaptic instability. Dysregulation of the mTOR/autophagy axis, observed in both MDS (Buist et al., 2022; Zha et al., 2019) and NS relative to Control CSF (with downregulation of YWHAB/Z, FKBP1A, eIF4G1, and ATP6V1A/B2/E1 subunits) (Sancak et al., 2010; Hoeffer et al., 2008; Pozuelo-Rubio, 2012; Fonseca et al., 2008), suggests that cells cannot effectively clear protein aggregates or maintain lysosomal acidity required for vesicular degradation.

These stress-adaptive states align with molecular features of neurodevelopmental arrest. In NS vs. Control CSF, the elevation of BRINP1, a BMP/retinoic acid-inducible protein, indicates a shift toward neuronal progenitor cell (NPC) quiescence, which may limit the expansion and maturation of neural circuits. This finding mirrors observations in brain organoid models of Rett syndrome and MDS, where *MECP2* dysfunction disrupts BMP signaling and NPC maintenance (Hong et al., 2023; Squillaro et al., 2012; Yang et al., 2024).

The MECP2- dependent regulation of *BDNF* provides a further mechanistic parallel. While *MECP2* loss-of-function in Rett syndrome reduces *BDNF* expression (Chapleau et al., 2009; Zhou et al., 2006; Sampathkumar et al., 2016; Kim et al., 2021), *MECP2* overexpression in MDS is frequently, though not universally, associated with increased BDNF (Li and Pozzo-Miller, 2014). In NS CSF (model excluding the NS5 outlier), the detected BDNF corresponds specifically to the precursor form (proBDNF), while NTRK2, the high-affinity receptor for mature BDNF (mBDNF), is concurrently elevated. While the absence of mBDNF in our dataset may be attributed to the inherent detection limits of shotgun proteomics for low molecular-weight proteins, the reciprocal elevation of NTRK2 suggests a state of trophic decoupling. In neurobiological systems. NTRK2 levels often fluctuate in response to ligand availability; its elevation in NS vs. Control CSF may reflect a compensatory upregulation or a failure in receptor internalization due to limited mature ligand engagement.

Importantly, proBDNF and mBDNF exert opposing biological effects. Whereas, mBDNF promotes synaptic stabilization and plasticity via NTRK2, proBDNF signals through p75NTR-sortilin complexes to drive synaptic pruning, apoptosis, and long-term depression (Teng et al., 2005). A milieu enriched in proBDNF alongside elevated NTRK2, without confirmed mBDNF, suggests a neurotrophic misalignment. In this state, the brain may be locked in a developmental arrest, where potential reparative cues are stalled at the precursor stage or misinterpreted as cues for elimination. Together, these findings delineate a shared trajectory characterized by NPC arrest, impaired MECP2-BMP coordination, and dysregulated neurotrophic signaling, culminating in the maladaptive synaptic integration and circuit instability common to Rett syndrome, MDS, and NS.

Developmental markers (e.g., RELN, ASTN2, BMP, WIF1, GPC6, and ROR1) were also elevated in NS vs. Control CSF. These molecules normally guide neuronal migration and cortical patterning (Filmus, 2022; Borcherding et al., 2014; Meyer et al., 2002; Alexander et al., 2023; Wilson et al., 2010; Parisi et al., 2020; Shimogori et al., 2004); however, their persistence in older NS individuals suggests a reactivation of developmental programs, potentially reflecting ongoing repair attempts that sustain immature circuitry and hinder cortical refinement.

At later stages, both NS and MDS seem to converge on tauopathy, yet with regionally distinct signatures: midbrain and cortical involvement in NS (Pollanen et al., 2018) vs. predominantly hippocampal pathology in MDS (Maphis et al., 2017; Vilvarajan et al., 2023). These differences likely reflect divergent timing and etiology: germline *MECP2* duplication in MDS perturbs hippocampal maturation from conception, whereas environmentally primed *MECP2* dysregulation in NS arises later, after hippocampal circuits have largely stabilized. This delayed onset may preferentially impact metabolically demanding midbrain and brainstem nuclei, which remain highly vulnerable during critical developmental windows.

Immune parallels are also notable. *MECP2* overexpression represses NF-κB and Th1 signaling (Yang et al., 2012; O'Driscoll et al., 2013), generating an immunosuppressive baseline (Boztug et al., 2016; Collette et al., 1996) consistent with recurrent infections in MDS (Van Esch, 2008; Bauer et al., 2015; Allison et al., 2024) and systemic tolerogenic vulnerability in NS. Clinically, this manifests as a notable susceptibility to filarial infections (Edridge et al., 2023) and elevated viral titers (Valdes Angues et al., 2022) in NS, suggesting a failure of immunological surveillance that permits chronic antigenic load. This blunted systemic state contrast sharply with Rett syndrome's pro-inflammatory phenotype, where *MECP2* loss-of-function drives NF-κB hyperactivation (Kishi et al., 2016).

Importantly, although NS aligns most closely with MDS, it also overlaps clinically with Rett syndrome, including regression, loss of ambulation, dyspraxic wandering, ataxia, dystonia, epilepsy, parkinsonian features, hypoactivity, and recurrent respiratory infections (Supplementary Table 2). Dysautonomic signs such as growth retardation and cold extremities are recognized in atypical Rett syndrome (Neul et al., 2010) and are likewise observed in MDS (Supplementary Table 2).

Collectively, these parallels support a shared pathophysiological axis centered on *MECP2* dysregulation. While MDS arises from germline *MECP2* duplication and Rett syndrome from *MECP2* loss-of-function mutations, we hypothesize that NS reflects an acquired environmentally primed disruption of the same regulatory axis. Despite distinct etiologies, all three conditions converge on destabilized neural trajectories, supporting a unifying framework in which *MECP2* imbalance, whether upward, downward, or environmentally modulated, drives overlapping neurodevelopmental and neurodegenerative phenotypes.

### 4.3 Prenatal priming: a plausible context

While NS typically presents in children aged 3 to 18, the proposed model posits that disease vulnerability may be seeded *in utero*, when maternal exposures, such as infection, malnutrition, biotoxins, or psychosocial stress, can reprogram fetal neural and immune systems through immunological and epigenetic mechanisms (Ratnayake et al., 2013; Kwon et al., 2022; Bilbo and Schwarz, 2009). In this context, MIA may act as a key initiator of two distinct yet convergent developmental trajectories.

First, MIA may imprint a tolerogenic bias in fetal glial lineages, particularly microglia, reducing their responsiveness to subsequent immune challenges (Otero and Antonson, 2022; Ostrem et al., 2024; Loayza et al., 2023; Mold et al., 2008; Fernandes and Lim, 2024; Amir and Zeng, 2021). Such immunological desensitization likely reflects an evolutionary trade-off: an adaptive response designed to protect the vulnerable fetal brain from collateral damage during maternal inflammation. While this imprinting may promote fetal survival by shielding the brain from inflammatory injury *in utero*, it establishes a postnatal mismatch in which neuroimmune surveillance and proteostatic responses remain blunted despite mounting cellular stress. The absence of a canonical acute-phase response, typically driven by glial activation and neurovascular signaling, suggests not a failure to respond *per se*, but rather a fundamentally altered damage recognition program.

Concurrently, MIA may induce epigenetic modifications, such as *MECP2* promoter hypomethylation, thereby predisposing multiple neural cells to *MECP2* overexpression (Basil et al., 2014). In neurons, where MECP2 is naturally high, excessive MECP2 levels can weaken excitatory transcriptional networks and impair lysosomal-autophagy capacity, with downstream effects on synaptic maturation and circuit formation (Basil et al., 2018; Ballas et al., 2009). In glial populations, including astrocytes and oligodendrocytes, elevated MECP2 may further suppress autophagy and metabolic support, exacerbating deficits in protein clearance and synaptic refinement (Ballas et al., 2009; Song et al., 2014; Jobe and Zhao, 2017).

Importantly, this blunted immune and proteostatic landscape often extends systemically beyond the BBB, potentially contributing to heightened vulnerability to environmental pathogens. Such an immunological profile may underlie the observed susceptibility of NS-affected children to nematode infections and viral persistence (Valdes Angues et al., 2022; Edridge et al., 2023), as attenuated surveillance can permit chronic bioenergetic drains and prolonged viral engagement that further destabilize *MECP2*-linked regulatory networks.

It is notable that this developmental vulnerability is not confined to *MECP2* alone. War-related trauma and gestational stress, pervasive within NS-prone regions, have been associated with methylation changes in other maternal and fetal genes, including *BDNF* (Kertes et al., 2017), itself a direct target of MECP2 (Larimore et al., 2009). Such epigenetic perturbations, whether acting on *MECP2* or its downstream effectors, may converge to increase postnatal susceptibility to a spectrum of environmental and metabolic stressors with neurotoxic potential.

Ultimately, prenatal priming of immune tolerance and *MECP2* dysregulation may establish a developmental trajectory in which synaptic pruning falter and proteostatic burden progressively accumulates (Hayes et al., 2021; Kodila et al., 2024; Kipnis, 2018; Ransohoff and Brown, 2012). Within this destabilized landscape, neuronal tau-driven degeneration becomes not merely a risk but a latent inevitability awaiting amplification. Postnatal exposures with tauogenic potential, including environmental biotoxins such as microcystin-LR (Section 4.6), may then act as secondary accelerants, transforming developmental fragility into overt neurodegenerative pathology.

### 4.4 Tiered and directional protein network analysis

Tiered network analysis in NS identified four dysregulated modules: coagulation, immune signaling, cytoskeletal remodeling, and stress adaptation. Overlaying directional information sharpened this architecture, exposing a dual signature: systemic suppression of core homeostatic processes alongside selective, but ultimately insufficient, adaptive responses.

Across all tiers, repression of complement, proteasome, cytoskeletal, autophagic, and mitochondrial modules reflects a collapse of the cellular scaffolding that underlies survival, synaptic function, and vascular integrity. This coordinated silencing suggests that NS pathophysiology is systemic, arising from a broad disruption of foundational processes rather than isolated deficits.

Coagulation proteins, particularly prominent in Tiers 1 and 2, provide a key example. Beyond their canonical role in hemostasis, these proteins (e.g., fibrinogen, plasminogen, kallikreins) are immune regulators and ECM remodelers. Their loss in NS likely promotes BBB vulnerability and maladaptive synaptic pruning, situating coagulation at the interface of peripheral stress and central neuronal fragility (Merlini et al., 2019; Dean et al., 2024; Ismail et al., 2021; Pepper, 2001).

In contrast, a subset of proteins, largely within Tier 2, was upregulated in NS relative to Control CSF, reflecting partial activation of stress-adaptive and remodeling programs. NLGN1 (Staab et al., 2014; Südhof, 2008) and ASTN2 (Ito et al., 2023) exemplify synaptic and circuit-level responses possibly linked to oxidative stress and disrupted connectivity, while VEGF-C points to vascular repair under hypoxic or redox challenge (Hossain et al., 2024; Liu et al., 1995). However, these compensatory efforts may be maladaptive: overexpression of synaptic organizers risks excitatory/inhibitory imbalance and elevated VEGF-C, while reparative, can sensitize cells to apoptosis under chronic stress.

Taken together, this pattern reflects a state of suspended equilibrium, where repression of core structural and metabolic systems coexists with incomplete, stress-driven compensatory responses. The recurrence of the same functional modules across tiers, and the consistency of their directional imbalance, underscores their role as nodal vulnerabilities, locking the system into chronic oscillation between progressive degeneration and partial, yet insufficient, recovery.

### 4.5 The stepwise collapse model of NS

**Figure 3:**
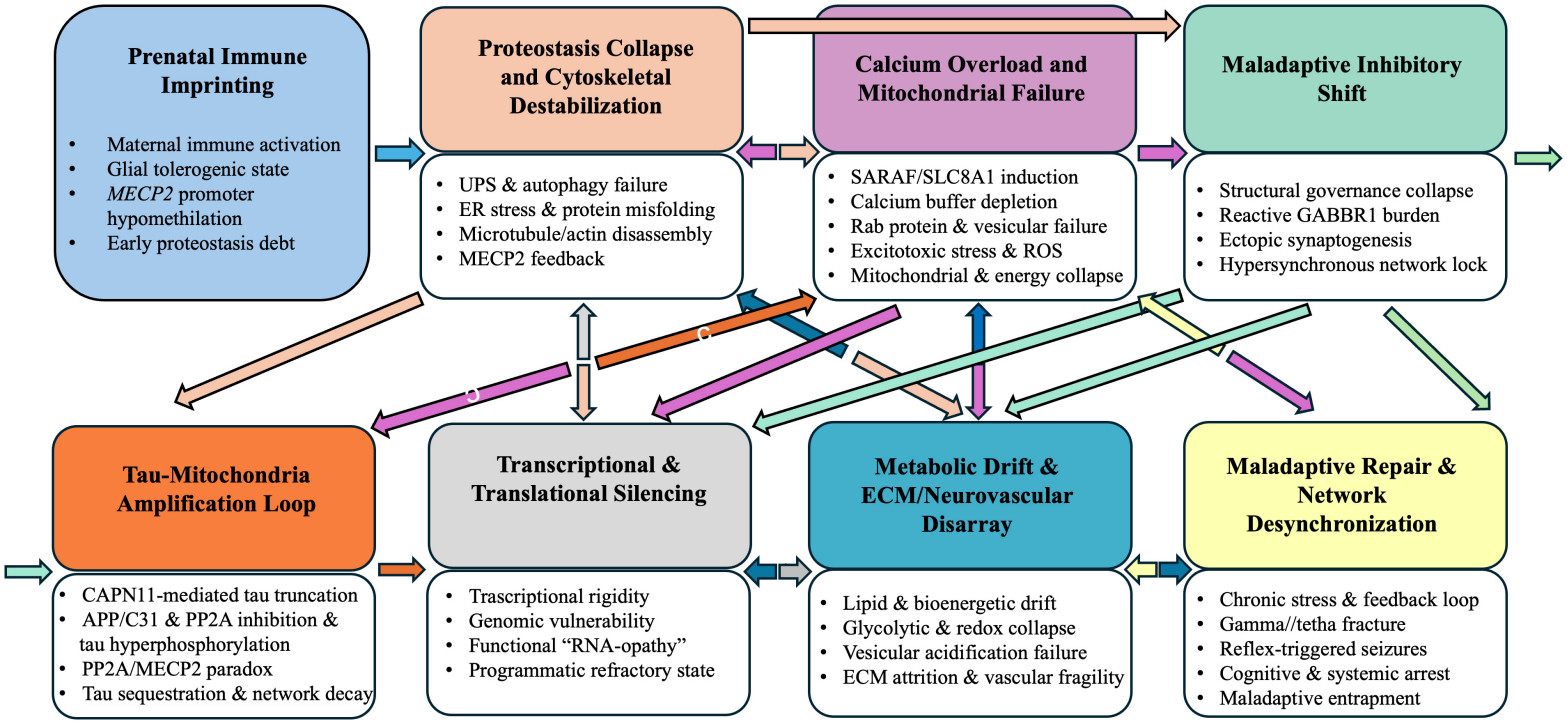
Stepwise, self-reinforcing molecular and network collapse in NS. The schematic summarizes molecular modules identified through CSF proteomic analysis, linking early immune and epigenetic perturbations to proteostatic failure, calcium and mitochondrial dysfunction, tau-associated processes, metabolic and neurovascular disarray, and maladaptive inhibitory network remodeling. Bidirectional arrows indicate potential recursive interactions among molecular modules. Although presented in a stepwise sequence for conceptual clarity, these processes are likely to occur simultaneously and synergistically. This framework represents a systems-level, hypothesis-generating model rather than a linear or singular causal pathway.

We propose that NS is a staged, self-reinforcing cascade, seeded by prenatal immune imprinting, amplified by proteostatic and cytoskeletal instability, and accelerated by mitochondrial stress and tau-mediated feedback loops, ultimately culminating in network desynchronization (Figure 3). At each stage, dysfunction recursively converges on a central destabilizing node, namely *MECP2* dysregulation, which integrates immune, metabolic, and synaptic failure. As outlined above, the prenatal phase (Section 4.3) may be triggered by MIA, while postnatal pathology (Section 4.6) likely reflects environmental amplification of this intrinsic vulnerability.

#### 4.5.1 Tolerogenic immune imprinting and proteostatic debt

The pathological cascade in NS may originate *in utero*, where MIA durably reprograms fetal glial progenitors toward a tolerogenic, hyporesponsive immunophenotype. When this imprinting occurs during critical windows of CNS maturation, it disrupts immune surveillance, dampens microglial process motility, and maladaptively resets long-term homeostatic set points essential for postnatal development (Ozaki et al., 2020; Kalish et al., 2021). Convergent epigenetic disruptions, such as *MECP2* promoter hypomethylation (Basil et al., 2014), may further compromise autophagic flux and lysosomal degradation (Zha et al., 2019), amplifying intracellular stress. The result is a proteostatic debt: a developmentally acquired burden of undegraded intracellular material (Choi and Chung, 2025) that progressively erodes synaptic pruning, antigen processing, and glial maturation (Cowan and Petri, 2018; Schafer et al., 2012; Janeway et al., 2001). Within this framework, early vulnerability stems not from overt inflammation but from a failure to sustain cellular homeostasis.

Proteomic profiling of NS CSF reinforces this model. Broad downregulation of innate and adaptive immune mediators, including acute-phase proteins (e.g., SAA1/2, sCD163, SERPINA3), complement components (e.g., C2, C4A, CFB, FCN3), chemotactic cues (e.g., S100A8/A9), and adaptive regulators (e.g., IGHM, PTPN6), indicates a globally suppressed immune landscape (Kipnis, 2018; Ransohoff and Brown, 2012; Ricklin et al., 2010). Diminished myeloperoxidase (MPO) supports this interpretation, reflecting blunted myeloid-cell activation and impaired responses to proteotoxic stress. Concurrent depletion of SET, a PP2A regulator (Dacol et al., 2021), and DDX39B, a key RNA helicase essential for maintaining inflammatory thresholds and NF-kB regulation (Hirano et al., 2023; Szymura et al., 2020), suggests a profound loss of immune plasticity, reflecting a systemic dismantling of stress-sensing and response pathways.

Yet, this is not passive immunodeficiency Select immune and developmental factors, including CD200, PRL, ROR1, C1QTNF4, and GPC6, are upregulated, indicating the persistence of fetal-like immunological programs that are misaligned with postnatal demands (Kipnis, 2018; Ransohoff and Brown, 2012). Increased expression of context-specific immunomodulators, such as SFTPD and FCER2, stands in stark contrast to the broad suppression of canonical antigen-processing and proteostasis machinery (e.g., PSME1, VCP, HSPA8, FKBP1A). This divergence signals an actively maintained tolerogenic state rather than a simple collapse of immune function.

Within this altered landscape, the NS CNS appears chronically desensitized yet unstable, permissive to metabolic and excitotoxic stressors but ineffective at clearance and repair (Bilbo and Schwarz, 2012). Dysregulated NF-κB signaling, in concert with atypical ROR1-mediated WNT5a activity, likely transforms early immune tolerance into a maladaptive feed-forward loop. In this state, immune hyporesponsiveness, proteostatic overload, and excitotoxic stress recursively amplify one another. The elevation of HLA-C, despite impaired antigen processing, suggests that MHC class I signaling is occurring via non-canonical pathways, decoupled from effective peptide presentation. This further confirms a functional disconnect: the brain signals an immune response, yet cannot enact it.

This framework reconciles the apparent paradox of NS as a condition marked by both systemic immune suppression and localized reactive gliosis. Canonical immune pathways remain broadly muted, reflecting a developmentally imprinted tolerogenic state. Yet, focal, maladaptive microglial activation emerges locally in response to discrete stressors, consistent with evidence that microglia, while tolerant at baseline, retain context-dependent reactivity (Neher and Cunningham, 2019; Towriss et al., 2023). The NS CSF signature, marked by suppressed NF-κB components, altered proteostasis regulators, and persistent developmental cues, captures this tension: not as immune protection but as chronic cellular injury masked as immune paralysis.

Collectively, these findings support a model of developmentally imprinted immune miscalibration: a tolerogenic framework rendered unstable. The machinery for repair exists, but operates out of synchrony with postnatal demands, masking injury until a metabolic threshold is breached. In this light, NS is not a classical inflammatory disorder but a misalignment of immune logic, a dissonance between developmental encoding and postnatal homeostatic needs.

#### 4.5.2 Proteostasis collapse and cytoskeletal destabilization

Once proteostatic capacity is compromised, neuronal dysfunction may extend beyond the accumulation of misfolded proteins and damaged organelles to directly undermine structural homeostasis. In neurons, quality-control systems are intimately coupled to cytoskeletal turnover; thus, sustained perturbations in proteostasis are expected to precipitate a structural collapse. In NS CSF, coordinated downregulation of the UPS (e.g., UCHL1, UBA1, UBE2K) and autophagy-lysosome pathway (e.g., HSPA8, VCP, HMGB1, PARK7) possibly reflects a failure to clear bulky cytoskeletal polymers, rendering neurons structurally brittle and functionally compromised.

Consistent with this observation, we report widespread dysregulation of microtubule and actin dynamics. Reductions in stabilizers like MAP2 and TUBA1A, alongside synaptic modulators like SNCA, suggest a shift from adaptive remodeling toward a structurally rigid, dysfunctional state. These deficits have profound consequences for axonal transport, dendritic plasticity, and synaptic architecture (Grant, 2012; Duran-Aniotz et al., 2022; Weng and He, 2021). Importantly, structural deterioration not merely follow proteostatic failure but may also contribute to broader network instability and reduced neuroplastic capacity (Dent et al., 2011; van der Kooij et al., 2016; Sheng and Pak, 1999).

Mechanistically, cytoskeletal fragility in NS points to a bioenergetic-structural crisis. Depletion of VCP and vacuolar H^+^-ATPase subunits likely impairs mitochondrial quality control (mitophagy), thereby reducing the ATP availability required for energy-intensive processes such as actin polymerization and microtubule-based transport. Simultaneously, aberrant epigenetic control, potentially mediated by dysregulated *MECP2* expression, may disrupt cytoskeletal gene programs, further weakening intracellular scaffolding (Pascual-Alonso et al., 2024, Section 4.5.6).

This destabilization possibly extends beyond the cell-intrinsic compartment. Concurrent degradation of ECM components, including fibrinogen chains, PLG, and MMPs, accompanied by loss of vinculin (VCL) and talin (TLN1), signifies a molecular disconnection from the extracellular support required for synaptic anchoring and repair. Because VCL and TLN1 form critical mechanical bridges between the actin cytoskeleton and the ECM, their reduction undermines structural continuity and mechanotransduction (Stamenkovic, 2003). Consequently, mechanosensitive signaling pathways such as NF-κB and Wnt may shift from roles in adaptive plasticity toward maladaptive signaling, further exacerbating oxidative stress and aberrant excitability (Jover-Mengual et al., 2021; Vallée et al., 2021; Vallée, 2022).

In sum, cytoskeletal collapse in NS may represent a critical juncture where proteostatic impairment is locked in, leading to progressive network dysfunction and a permanent loss of neural resilience.

#### 4.5.3 Calcium overload and mitochondrial failure

As structural integrity erodes, both within neurons and their extracellular interfaces, the ability to preserve ionic and metabolic homeostasis may become increasingly compromised. Loss of core cytoskeletal stabilizers (e.g., MAP2, TUBA1A, spectrins) and anchoring proteins (e.g., VCL, TLN1) in NS CSF points to a breakdown in the spatial organization of calcium transporters. This structural decay is likely compounded by proteostatic failure, where the reduction of VCP and UPS components impedes turnover of damaged ion channels, further destabilizing membrane function.

Consistent with these structural deficits, NS CSF exhibits selective upregulation of the sodium/calcium exchanger SLC8A1 and the SOCE regulator SARAF, indicating a reactive attempt to restore calcium homeostasis by promoting cytosolic calcium efflux and limiting further influx (Kodakandla et al., 2023; Dagan and Palty, 2021; Lytton, 2007). However, this membrane-level compensation appears compromised by reduced intracellular calcium buffering (e.g., CALB1, CALM1, TPT1) and persistent loss of cytoskeletal scaffolds critical for channel regulation (MAP2/TUBA1). Without effective sequestration and retention, increased intracellular calcium may persist, activating calcium-dependent proteases (e.g., calpains) and initiating a feed-forward loop in which excitotoxic signaling and structural degradation recursively amplify one another (Verma et al., 2022; Choi, 1988; Lau and Tymianski, 2010).

Compounding these ionic disturbances, the neuronal capacity to neutralize calcium-induced oxidative stress appears critically compromised. Key antioxidant defenses, including the mitochondrial chaperone PARK7, essential for Complex I stabilization and ROS detoxification (Zhang et al., 2016, 2021), alongside catalase (CAT) and peroxiredoxins (PRDXs) (Nandi et al., 2019, 2016; McBean et al., 2015), are depleted. This collapse in redox buffering coincides with downregulation of oxidative phosphorylation components, such as COX6B1 and ECI1, signaling a broader decline in mitochondrial respiratory efficiency (Rottenberg and Hoek, 2021; Görlach et al., 2015; Nicholls, 2004).

Disruption of endosomal dynamics and Rab-mediated trafficking further exacerbates these metabolic vulnerabilities. Downregulation of RAB5C, RAB11A, and the Rab GDP-dissociation inhibitor RABGDIB suggests a systemic collapse of the endocytic-lysosomal axis. RABGDIB, a master chaperone for retrieving Rab GTPases from membranes (Müller and Goody, 2018), is particularly vital for RAB11A recycling, a preferential GDI target with essential roles in membrane trafficking (Shafique et al., 2023). This convergent breakdown in Rab regulation imposes a critical bottleneck: without effective Rab recycling, neurons cannot shuttle damaged mitochondria to lysosomes for mitophagy or reallocate calcium transporters, leaving the cell trapped in a state of organelle congestion, functional stagnation, and progressive decay.

These ionic, oxidative, and trafficking deficits are likely amplified by MECP2-driven transcriptional dysregulation and aberrant Wnt-ROR1-calcium signaling, which may further enhance calcium influx and divert stress responses toward degenerative pathways (Signorini et al., 2016). Concurrent suppression of NF-κB mediated clearance may also preclude activation of essential DNA repair programs (Section 4.5.6) and autophagic turnover of damaged proteins (Section 4.5.2), creating a compounding cycle in which unrepaired genomic lesions and collapsing structural scaffolds reinforce one another.

Together, these interconnected disruptions position NS along a pathological continuum that shares mechanistic features with classical neurodegenerative disorders, including AD (Calvo-Rodriguez and Bacskai, 2021; Bhatia et al., 2022) and parkinsonian syndromes (Moon and Paek, 2015). Mitochondrial Complex I vulnerability, a hallmark of Parkinson's disease (PD), often arises from oxidative damage and functional deficiency (Keeney et al., 2006; Schapira et al., 1990), a state that chronic calcium elevation and ROS accumulation could similarly induce in NS. However, the accompanying immunological profile reveals a critical distinction. In PD-like pathologies, microglial activation typically drives upregulation of TREM2, a key myeloid receptor that senses lipid-rich debris and orchestrates the phagocytic clearance of damaged neurons (Zhidan et al., 2025). By contrast, TREM2 is downregulated in NS CSF (model excluding the NS5 outlier), consistent with a globally immunosuppressive or tolerogenic phenotype. This divergence underscores NS as a distinct hybrid pathology, one that may be neurodevelopmentally primed toward immune silencing and subsequently rendered susceptible to progressive metabolic and structural decay.

Rather than being confined to the neuronal compartment, this decay signals a systemic collapse of the broader neuroglial ecosystem. While neurons are particularly vulnerable due to their high energetic and structural demands, the widespread depletion of proteostatic and metabolic proteins indicates a concurrent failure of glial support. Loss of astrocytic metabolic buffering and impaired microglial surveillance, evidenced, for instance, by downregulation of TREM2, plausibly compounds intrinsic neuronal vulnerability. Within this deteriorating framework, the neuron does not fail in isolation; rather, it likely collapses as escalating homeostatic demands exceed the waning support of the surrounding glial network.

#### 4.5.4 Maladaptive inhibitory shift

Seizure activity is traditionally attributed to an imbalance favoring excitatory over inhibitory neurotransmission. However, in neural systems chronically exposed to calcium overload and mitochondrial failure (Section 4.5.3), inhibitory compensation can itself become maladaptive.

In NS CSF, the signature of excitatory synaptic destabilization is defined by a profound loss of structural governance. Downregulation of key cytoskeletal scaffolds (namely MAP2 and SNCA) indicates a collapse of the framework essential for glutamatergic integrity. This architectural failure is exacerbated by sustained calcium overload (Section 4.5.3), which recruits calcium-dependent proteases (e.g., calpains) that further dismantle the postsynaptic density. Simultaneously, impaired clearance pathways (Section 4.5.2) allow for the accumulation of these degradation products and the persistence of damaged ion channels. Unanchored, unregulated ion channels sustain a state of chronic calcium influx, creating a self-reinforcing loop of structural disintegration. Within this failing architecture, the elevation of GABBR1 likely reflects a reactive effort to mitigate excitotoxicity (Chalifoux and Carter, 2011), rather than a globally hypoexcitable state. However, this inhibitory burden appears maladaptive, likely suppressing nascent network activity without resolving the underlying ionic derangement. This state may be further entrenched by MECP2-mediated suppression of excitatory plasticity (Cronk et al., 2017), which limits synaptic reintegration and effectively locks the circuit into a non-functional degenerative trajectory.

Concurrently, upregulation of synaptic organizers (e.g., NLGN1/3, RELN, TNR) and the axon guidance protein SEMA6A suggests an attempt to reestablish excitatory connectivity, a process typically associated with adaptive remodeling following acute injury (Rogalewski et al., 2010). However, in the chronic excitotoxic environment of NS (Hoffe and Holahan, 2022), and in the absence of stable anchoring proteins like VCL and TLN1 (Section 4.5.2), these remodeling efforts may culminate in ectopic synaptogenesis, a structural organization devoid of functional engagement. This architectural shift may foster a self-reinforcing excitatory/inhibitory disequilibrium, wherein excitotoxic injury prompts reactive inhibition that, in turn, constrains the potential for excitatory recovery.

By physically stabilizing maladaptive circuits within a rigid, pathologically altered extracellular and cytoskeletal scaffold (Dankovich and Rizzoli, 2022), the NS brain may become locked into a hypersynchronous, low-variability state. This failure of synaptic scaling and neuronal plasticity can establish a seizure-prone network architecture, a phenotype shared with ASD and *MECP2*-overexpression syndromes such as MDS (Nguyen et al., 2020).

#### 4.5.5 The tau-mitochondria amplification loop

As structural and excitotoxic stress accumulates in NS, the system may cross a pathological threshold beyond which damage becomes self-reinforcing. At the core of this transition is a reciprocal amplification loop between mitochondrial dysfunction and tau dysregulation.

Sustained calcium overload (Section 4.5.3) activates calcium-dependent proteases, namely CAPN11. Although classical calpains (CAPN1/2) are prominent mediators of proteolysis in typical neurodegeneration, the selective elevation of the non-canonical CAPN11 in NS CSF suggests an atypical proteolytic profile; one that may facilitate generation of neurotoxic aggregation-prone tau fragments and accelerate degradation of cytoskeletal scaffolds (Ferreira and Bigio, 2011; Metwally et al., 2021, 2023).

This aberrant proteolysis plausibly synergizes with the described proteostatic debt (Section 4.5.1). The cell can neither prevent tau truncation nor engage integrated clearance pathways to degrade the pathogenic fragments. Truncated tau species can associate with the mitochondrial outer membrane, disrupting oxidative metabolism, as evidenced by downregulation of COX6B1 (Complex IV) and ECI1 (β-oxidation). Loss of respiratory integrity and metabolic flexibility likely precipitates mitochondrial depolarization, potentially triggering mitochondrial permeability transition pore (mPTP) opening (Quintanilla et al., 2009) and subsequent ATP depletion. The resulting energy crisis and ROS accumulation may further activate stress-induced kinases such as GSK-3β and proteases like CAPN11, propagating a vicious cycle of tau truncation and hyperphosphorylation.

Concurrently, aberrant APP processing in NS CSF yields C31 fragments (Nishimura et al., 2002; Park et al., 2020; Lu et al., 2003; Chen et al., 2024; Furgerson et al., 2012) that inhibit PP2A, the primary phosphatase responsible for tau dephosphorylation (Park et al., 2012; Qian et al., 2010). Because PP2A also restrains tau-kinases, its inhibition unleashes GSK-3β activity, skewing tau toward a hyperphosphorylated state (Qian et al., 2010; Kim et al., 2003). The combination of CAPN11-driven truncation and PP2A inhibition with GSK-3β activation, creates a biochemical milieu in which truncated tau seeds aggregation while remaining soluble tau becomes hyperphosphorylated and functionally inert.

The resulting collapse in tau solubility may facilitate sequestration of mixed 3R/4R tau isoforms into insoluble assemblies. Unlike the gradual maturation of neurofibrillary tangles typical of AD (Sayas and Ávila, 2021; Dregni et al., 2022; Therriault et al., 2022), the metabolic failure in NS likely favors the formation of pre-tangles and dot-like grains, morphologies that match neuropathological findings in post-mortem NS brains (Pollanen et al., 2018; Pollanen and Onzivua, 2023). This pattern reflects an overwhelmed proteostatic system, in which misfolded tau accumulates faster than chaperone networks can resolve (Klaips et al., 2018). Widespread depletion of tau-stabilizing and cytoskeletal support proteins (e.g., MAP2, SNCA, YWHAQ, HSP90AA1) (Sluchanko and Gusev, 2011) in NS CSF, further suggests that both the structural architecture and the proteostatic machinery of the neuron have reached a breaking point.

Within this destabilized landscape, the PP2A-MECP2 axis presents a critical regulatory paradox. While PP2A inhibition (driven by C31 fragments) may transiently lower MECP2 levels, potentially ameliorating select MDS-like phenotypes (Lombardi et al., 2017), it simultaneously accelerates tau hyperphosphorylation and aggregation (Sontag et al., 1999). Attempts to restore PP2A activity to dephosphorylate tau, however, may stabilize MECP2, which in turn suppresses autophagy (Zha et al., 2019) and halts clearance of aggregated tau species (Sontag et al., 1999). Consequently, any reactive homeostatic effort to recruit PP2A for tau dephosphorylation may inadvertently stabilize MECP2-linked repressive programs. This bidirectional constraint creates a regulatory bottleneck: normalization of one substrate (tau) potentially exacerbates the dysregulation of the other (MECP2), effectively locking the neuron into a state of proteostatic stasis.

This bottleneck further distorts tau clearance dynamics, decoupling parenchymal burden from CSF dignaling. By preventing tau diffusion into the extracellular space, parenchymal sequestration likely underpins the paradoxical dissociation between NS tissue pathology and normal-range CSF tau levels, a signature distinct from AD, where both total and phosphorylated tau are typically elevated (Blennow et al., 2010; Lantero-Rodriguez et al., 2024; Milos et al., 2024).

Primary tauopathies characterized by high-affinity sequestration, such as progressive supranuclear palsy (PSP) and corticobasal degeneration (CBD), reinforce this model. Despite heavy parenchymal burden, CSF tau levels often remain low (Ishiguro and Kasuga, 2024; Giagkou et al., 2024; Giannakis et al., 2025), indicating that CSF tau reflects solubility and clearance dynamics rather than total load. In NS, the clearance failure imposed by the PP2A-MECP2 axis, likely amplifies this dissociation, rendering the 3R/4R tau pool functionally insoluble. The resulting tau grain morphologies (Pollanen et al., 2018; Pollanen and Onzivua, 2023) are likely to evade standard detection, resistant to untargeted MS due to poor tryptic coverage, ion suppression, and low soluble stoichiometry (Smith and Rogowska-Wrzesinska, 2020; Olsen and Mann, 2013).

In sum, normal-range CSF tau does not exclude significant tauopathy in NS; instead, it reflects a distinct pathological signature shaped by mitochondrial dysfunction, impaired proteostasis, and surveillance failure. Atypical tau fragments likely remain sequestered within the parenchyma, failing to access CSF clearance routes. Under typical neurodegenerative conditions, such species would engage TREM2-mediated phagocytosis; however, in NS, TREM2 downregulation implies failure of this surveillance pathway, permitting persistence of proteotoxic burden.

#### 4.5.6 Inhibitory arrest and transcriptional silencing

As oxidative stress and metabolic failure deepen (Section 4.5.3), neurons in NS appear to enter a progressively suppressed transcriptional and translational state (Akiyama and Ivanov, 2024; Kreuz and Fischle, 2016). This regulatory shutdown disrupts the entire gene-expression cascade, from RNA biogenesis and splicing to protein synthesis and genomic surveillance. Our CSF proteomic data reveal a significant depletion of critical nucleotide metabolism enzymes (PAICS, PNP, AHCY), indicative of limited nucleotide availability and compromised methyltransferase capacity (Vizán et al., 2021; Fukumoto et al., 2022). Such deficits likely impair chromatin regulation, a domain in which MECP2 plays a central role (Fasolino and Zhou, 2017; James et al., 2002). Furthermore, the reductio of TALDO1, a core enzyme in the non-oxidative branch of the PPP, creates a metabolic bottleneck for nucleotide synthesis, directly undermining both DNA-repair capacity (Qian et al., 2008) and the energetically demanding process of transcription.

Superimposed upon this metabolic fragility, *MECP2* dysregulation may promote a state of transcriptional rigidity (Fasolino and Zhou, 2017; James et al., 2002; Boxer et al., 2020; Kinde et al., 2016), limiting the adaptive plasticity of neuronal gene programs. This rigidity is likely exacerbated by declines in DNA-repair sensors and effectors (e.g., XRCC5, APEX1, RPA1), which facilitate the recognition and resolution of oxidative lesions and double-strand breaks within transcriptionally active, identity-defining genomic loci (Bai et al., 2025). While this transcriptional restraint might initially serve to mitigate further genomic instability, its persistence likely entraps the neuronal-glial ecosystem in a quasi-dormant regulatory state that is poorly responsive to metabolic or physiological challenges.

This pattern of regulatory arrest mirrors the regressive phenotypes observed in syndromes associated with perturbations in core RNA processing factors, such as SRRM2- (Cuinat et al., 2022) and DDX39B-associated (Booth et al., 2025) disorders. In these conditions, splicing and RNA trafficking defects precipitate seizures, developmental regression, and progressive neuronal loss. Notably, pathogenic DDX39B variants recapitulate multiple features reminiscent of NS, including developmental delay, hypotonia, epilepsy, skeletal anomalies, and dysmorphism (Booth et al., 2025). The synergistic downregulation of splicing factors (SRSF2, PTBP1) and RNA export mediators (DDX39B, SRSF1) in NS is consistent with a “functional RNA-opathy,” in which systemic failure to process and export transcripts leads to a permanent loss of cellular identity and transcriptional plasticity. This emergent phenotype effectively silences dynamic gene regulation, possibly entrenching both neurons and glia in a refractory state that precludes the capacity to adapt to stress, respond to injury, or restore homeostasis.

#### 4.5.7 Metabolic drift and collapse of structural integrity

Proteomic profiles from NS CSF also reveal a systemic failure across interconnected metabolic networks, consistent with a progressive metabolic drift rather than isolated pathway deficits. Perturbations spanning lipid, carbohydrate, and nucleotide metabolism may undermine cellular processes essential for detoxification, vesicular trafficking, and membrane turnover. Reduced availability of ATP and NADPH, central determinants of cellular energy and redox capacity, likely amplifies neuronal vulnerability and reinforces upstream stressors, including proteostatic failure, transcriptional repression, and mitochondrial dysfunction.

##### 4.5.7.1 Lipid metabolic drift and the endolysosomal bottleneck

Proteomic profiles from NS CSF revealed widespread dysregulation of lipid metabolism, reflecting systemic deficits in both degradation and synthesis. Enzymes central to fatty acid β-oxidation, including ECI1 (mitochondrial) and ACOX1 (peroxisomal), are markedly downregulated. This reduction, coupled with diminished aldehyde-detoxifying enzymes (e.g., AKR1A1/B1, ALDH1A1), points to systemic failure in lipid catabolism. The inability to effectively break down fatty acids likely limits energy availability and permits the accumulation of membrane-damaging lipid peroxidation byproducts (Lan et al., 2024; Dalleau et al., 2013; Trares et al., 2022).

In parallel, expression of ATP-citrate lyase (ACLY), a critical enzyme linking carbohydrate metabolism to *de novo* lipogenesis (Biju et al., 2024), is reduced. ACLY catalyzes the conversion of citrate into cytosolic acetyl-CoA, a key substrate for fatty acid and cholesterol synthesis (Wellen et al., 2009). In neurons, which rely heavily on continuous lipid supply to maintain and remodel the expansive surface area of dendritic and axonal membranes, ACLY deficiency is likely to impose structural vulnerability. This mechanistic link between metabolic insufficiency and membrane degeneration may underlie the cortical atrophy and white matter rarefaction observed in NS brain tissue (Pollanen et al., 2018, 2023; Pollanen and Onzivua, 2023).

This broad lipid metabolic exhaustion appears compounded by deficits in vesicular acidification. Multiple subunits of the vacuolar-type H?-ATPase complex (namely ATP6V1A, ATP6V1B2, and ATP6V1E1), are downregulated. This proton pump maintains the acidic environment of endosomes, lysosomes, and synaptic vesicles, enabling neurotransmitter loading (e.g., glutamate, GABA, dopamine), lysosomal proteolysis, and autophagic flux (Falace et al., 2024). Among these, ATP6V1B2 is a brain-enriched isoform critical for synaptic vesicle acidification. Its depletion has been implicated in syndromes featuring refractory epilepsy and cognitive regression (Falace et al., 2024; Kortüm et al., 2015), suggesting a potential pathogenic role in NS.

Acidification failure, in conjunction with ATP depletion, may further compromise proteostasis. ATP not only fuels cellular processes but also acts as a hydrotrope, maintaining protein solubility by preventing aberrant aggregation. Its reduction increases the risk of tau and other misfolded proteins forming insoluble complexes (Sarkar et al., 2024). Together, these disruptions in lipid metabolism, energetic supply, and lysosomal function converge to promote widespread synaptic vulnerability and structural decay.

Within this degenerative landscape, the upregulation of enzymes involved in sphingolipid and extracellular lipid metabolism (e.g., ASAH1, GALC, NPC2, CEL) may represent a compensatory response to persistent membrane stress. However, in the absence of restored ACLY-mediated lipogenesis and β-oxidative capacity, this response may prove insufficient to reestablish lipid homeostasis, ultimately accelerating neuropil destabilization and neuronal decline.

##### 4.5.7.2 Carbohydrate metabolism failure

A parallel and coordinated failure of carbohydrate metabolism is evident through downregulation of key enzymes across glycolysis, gluconeogenesis, and glycogenolysis, including ENO1, PKM, PGAM1, ALDOB/C, PGK1, GPI, and the glycogen phosphorylase PYGL. This broad suppression impairs both immediate glycolytic ATP generation and the mobilization of glycogen reserves (Johnson et al., 2020; Ryu et al., 2021), compounding energetic stress within vulnerable neuronal circuits.

This primary energetic deficit is further intensified by redox collapse. Depletion of IDH1, the brain's main cytosolic source of NADPH (Biedermann et al., 2019), alongside reduced expression of PPP enzymes (e.g., PGD and TALDO1), signals a critical failure to sustain the reducing environment required for glutathione recycling and oxidative defense.

The resulting redox instability heightens vulnerability to oxidative damage, while the underlying ATP crisis dismantles cellular systems responsible for maintaining protein solubility. Failure of ATP-dependent vacuolar pumps (V-ATPases) disrupts vesicular acidification (Section 4.5.7.1), stalling endo-lysosomal clearance of misfold proteins. Furthermore, declining ATP levels eliminate its intrinsic hydrotropic buffering capacity, promoting irreversible protein insolubility (Sarkar et al., 2024) and perpetuating pathogenic cascades, such as the tau-mitochondria amplification loop.

These proteomic signatures echo patterns observed across primary neurodegenerative disorders, where early cerebral glucose hypometabolism precedes overt neuronal loss on positron emission tomography (PET) imaging (Hammond and Lin, 2022). In NS, such metabolic exhaustion likely arises upstream of irreversible degeneration, imposing a bioenergetic constraint that limits neuronal capacity for both functional output and structural repair.

##### 4.5.7.3 ECM attrition and neurovascular fragility

As intracellular energy reserves decline (Sections 4.5.7.1 and 4.5.7.2) and cytoskeletal scaffolds deteriorate (Section 4.5.2), downstream consequences emerge in the extracellular milieu. Metabolically demanding structures, such as perineuronal nets (PNNs), become increasingly unsustainable under chronic energy stress (Wang and Fawcett, 2012; Rowlands et al., 2018). Consistently, NS CSF exhibits profound depletion of ECM proteins and vascular stabilizers (e.g., fibrinogen chains, PLG, KLK, ANXA5), along with downregulation of MMP3/8/9, suggesting a failure of ECM remodeling, essential for synaptic anchoring and BBB integrity (Stamenkovic, 2003; Zenaro et al., 2017; McConnell et al., 2017).

This structural decline extends to the neurovascular unit, reflected in the loss of multiple coagulation and hemostatic factors (FGG, F5, F9, F11, F13A1), potentially increasing vulnerability to micro-hemorrhages and cerebrovascular dysregulation. Although compensatory signals are evident, including increased synaptic stabilizers (e.g., NLGN1/3, ASTN2), angiogenic markers (VEGF-C) (Hossain et al., 2024; Lee et al., 2025) and pro-inflammatory mediators (TNFS12) (Khan et al., 2025), these responses unfold within a structurally compromised and energetically depleted system. Crucially, concurrent downregulation of core proteostatic effectors, such as HSPA8, indicates that attempted repair lacks chaperone-mediated support and cannot be sustained.

This convergence of metabolic failure, cytoskeletal degradation, ECM disintegration, and vascular fragility, reflects a unified collapse across intracellular and extracellular domains. Similar molecular signatures are observed in neurodevelopmental disorders such as ASD (Liu et al., 2021; Guldiken et al., 2024) and Rett syndrome (Pecorelli et al., 2020), where chronic mismatch between compensatory signaling and repair capacity underlies progressive dysfunction. In NS, this pattern may define a maladaptive homeostatic state; one in which structural and energetic fragility drives system-wide destabilization.

#### 4.5.8 Network desynchronization

At the systems level, the cumulative impact of recursive, interdependent failures undermines the brain's capacity to sustain coherent neuronal timing and circuit integration. Rather than arising from discrete focal lesions, this convergence is expected to produce widespread disruption of rhythmic neural activity, including gamma/theta decoupling and loss of long-range synchrony. Such network-level desynchronization reflects a progressive erosion of temporal precision and integrative capacity across distributed circuits (Deng et al., 2024), a state that cannot be attributed to excitotoxic injury alone. Clinically, this profile aligns with the emergence of atonic, reflex-triggered seizures, and cognitive arrest in NS, consistent with a model wherein developmentally constrained, *MECP2*-associated dysfunctions recursively destabilize higher-order network architecture.

Crucially, the proteomic signatures described herein represent a cross-sectional capture of NS pathophysiology, exposing a convergent vulnerability profile rather than a linear progression. While presented in stages for clarity, this model assumes these failures unfold simultaneously and recursively, reinforcing one another through shared energetic and regulatory constraints. This recursive breakdown results in a self-perpetuating collapse of cellular homeostasis, in which diminishing temporal coherence restricts neurodevelopmental plasticity, impairs recovery, and accelerates systemic entrapment.

Together, these findings support a unified model of NS as a developmentally primed systems disorder, characterized by structural fragility, proteostatic exhaustion, and network desynchronization, culminating in a terminal state of physiological and functional arrest.

### 4.6 Postnatal acceleration: environmental triggers

The postnatal environment may serve as a critical amplifier of pre-existing immune imprinting and *MECP2*-axis vulnerability, accelerating disease progression and shaping clinical manifestation. Chronic exposure to environmental neurotoxins, such as microcystin-LR from cyanobacterial blooms and ochratoxin A from contaminated food sources, has been implicated in BBB disruption, increased proteostatic burden, and potentiation of excitotoxic cascades (Spencer et al., 2024).

At the molecular level, microcystin-LR inhibits PP2A, promoting tau hyperphosphorylation and potentially altering MECP2 phosphorylation dynamics and protein stability (Liang et al., 2011). In parallel, ochratoxin A downregulates *MECP2* expression via upregulation of microRNA-132 (De Santis et al., 2019). While each of these mechanisms could independently yield Rett-like features, their convergence within a prenatally primed neural environment may produce phenotypic outcomes distinct from classical Rett syndrome, reflecting context-dependent modulation rather than pure *MECP2* loss-of-function.

Importantly, vulnerabilities seeded during gestation may remain latent until unmasked by critical neurodevelopmental transitions. In MIA models, for example, the loss of PNNs in prefrontal and limbic circuits emerges only after circuit maturation reaches functional thresholds, often during early adulthood (Paylor et al., 2016). Given that human PNN maturation spans from childhood through adolescence, this delayed vulnerability aligns with the typical onset window (3–18 years) of atonic seizures in NS.

Within this framework, postnatal exposure to neurotoxic agents may act on an already developmentally compromised system, accelerating the emergence of overt neurological dysfunction. Rather than redefining disease identity, such environmental triggers may modulate disease tempo, penetrance, and clinical severity, transforming latent molecular and circuit-level fragility into symptomatic expression.

### 4.7 Phenotypic variability and maternal reality

In NS-prone regions, maternal exposures to environmental agents rarely occur in isolation. Infectious burden, malnutrition, psychosocial stress, and toxin exposure frequently co-occur, creating a chronically pro-inflammatory gestational milieu (Monk et al., 2019; Zawadzka et al., 2021; Creisher et al., 2024; Ekregbesi et al., 2025; Bucknor et al., 2022; Tobi et al., 2015). Rather than producing a uniform developmental outcome, this convergence of stressors is likely to induce a spectrum of neurodevelopmental perturbations, with offspring phenotypes shaped by timing, intensity, and combinatorial impact of *MECP2*-axis disruption, immune imprinting, and synaptic sensitivity.

Phenotypic heterogeneity in NS may thus reflect both temporal dynamics and cumulative environmental load. Insults occurring during early windows of maximal epigenetic plasticity may establish enduring vulnerabilities, whereas later gestational or early postnatal exposures may modulate disease onset, severity, and/or progression (Collins et al., 2024; Bale et al., 2010). Postnatal accelerants, such as biotoxin exposure or infections, may act upon these prenatal imprints, offering one explanation for both familial clustering (e.g., shared maternal environments) and marked individual variability (e.g., stochastic overlaps across the prenatal-postnatal continuum) in NS presentation.

Maternal exposures may also recalibrate the gut-brain axis (Tartaglione et al., 2022), an increasingly recognized contributor to neurodevelopmental disorders and a proposed modifier in NS (Arony et al., 2018a,b). Both MIA and *MECP2* dysregulation are known to alter host-microbiota signaling, neuroinflammatory tone, and metabolite availability. In rodent models, MIA induces ASD-like behaviors alongside gut dysbiosis and cytokine shifts (Tartaglione et al., 2022), while *MECP2* overexpression in MDS similarly disrupts microbial composition and metabolic homeostasis (Wu et al., 2024).

Within this broader landscape, reproductive parity emerges as a potentially underappreciated modifier of NS risk. Epidemiological data show higher incidence of NS among first-, second-, and third-born children, with notable clustering among firstborns (Lagoro and Arony, 2017). Mechanistically, firstborns display reduced anti-inflammatory T cell responses at birth (Kragh et al., 2016), consistent with differences in *in utero* immune programming. Parity-associated epigenetic signatures, including shifts in DNA methylation and histone acetylation in immune and neuroplasticity genes, have also been documented (Campagna et al., 2023). Later-born children may benefit from early-life immune training via sibling-associated microbial exposures, which correlate with reduced allergy and inflammatory disease risk (Lisik et al., 2023a,b). First pregnancies, conversely, are characterized by distinct maternal cytokine and glucocorticoid patterns, potentially imprinting epigenetic marks in fetal microglia and neurons that bias immune tolerance and constrain synaptic pruning (Glover et al., 2018).

Taken together, these observations support a model in which NS represents not a monolithic entity but a spectrum of phenotypic expression, shaped by maternal priming across infection, nutrition, stress, parity, and microbiota. Within this landscape, firstborn offspring, bearing a comparatively immature immuno-epigenetic imprint, may be particularly vulnerable when postnatal environmental triggers further erode systemic resilience, accelerating progression along the NS cascade.

### 4.8 Nutritional resilience as a potential protective factor

Epidemiological patterns of NS prevalence, particularly its absence in certain populations, suggest that nutritional status plays a critical role in modulating physiological resilience along the *MECP2* axis. Notably, South Sudanese itinerant Dinka cattle herders and abducted Ugandan children have shown relative resistance to NS (Spencer, 2023; Tumwine et al., 2012; Landis et al., 2014). In contrast, Moru subsistence farmers in NS-affected regions of southern Sudan reportedly described the disorder as *Adravu Legnaro*, “a disease from eating *ugali*,” a maize- or sorghum-based porridge staple. Dinka communities, characterized by prolonged breastfeeding and diets rich in animal-derived foods (e.g., milk, meat, blood), remained largely unaffected (Spencer et al., 2013a). This contrast suggests that animal-based nutrition may confer metabolic buffering capacity, while monotonous agrarian diets may amplify underlying vulnerabilities (Mattson and Arumugam, 2018; Benton, 2010).

Plant-based subsistence diets dominated by maize, sorghum, and legumes also carry elevated risks of mycotoxin exposure and phytate accumulation, both of which can impair mineral absorption (Gupta et al., 2015). Chronic high phytate intake, particularly when unbuffered by animal protein, inhibits zinc and iron uptake, leading to micronutrient depletion. These deficiencies can deepen vulnerabilities seeded by prenatal immune stress. Zinc deficiency, in particular, has been linked to impaired immune competence, reduced synaptic stability, and heightened seizure susceptibility through excitotoxic mechanisms (Sandström, 2001; Brown et al., 2004).

Interventional data further support the role of nutrition as a modifiable protective factor. Early dietary stabilization, including access to varied, nutrient-dense local foods and multivitamin supplementation, has been associated with reduced seizure frequency and marked improvement in rehabilitation outcomes among NS-affected children, especially when combined with anti-seizure medication (Spencer et al., 2022; Gazda and Kitara, 2018; Idro et al., 2014). These findings are consistent with the view that *MECP2*-destabilized circuits remain metabolically fragile yet responsive to energetic and micronutrient repletion, even in the presence of established pathology.

Biochemical profiling of NS cohorts adds mechanistic support to this model. Elevated plasma levels of vitamins A and E, alongside reduced vitamin B12 (Edridge et al., 2023), suggest a pattern of compensatory antioxidant upregulation against a backdrop of metabolic stress. Vitamins A and E support redox homeostasis (Blaner et al., 2021), while vitamin B12 is essential for one-carbon metabolism and methylation. Vitamin B12 deficiency reduces S-adenosylmethionine availability, alters m6A mRNA methylation, and promotes hypomethylation of MECP2 target promoters, potentially exacerbating transcriptional instability (Mosca et al., 2021; An et al., 2019; Uekawa et al., 2009). Consistent with the nutrient sensitivity of this axis, experimental models demonstrate that perinatal high-fat diets can modulate *MECP2* expression in region- and sex-specific patterns, reshaping both metabolic programming and behavioral outcomes (Frayre et al., 2021).

Collectively, these convergent lines of evidence position nutrition not as a peripheral modifier but as a central regulator of *MECP2*-axis stability, with the capacity to buffer against, or exacerbate, seizure-prone neurodevelopmental trajectories.

### 4.9 Therapeutic insights

Ivermectin, long deployed as an antiparasitic agent and central to the OV hypothesis of NS etiology (Colebunders et al., 2017a,b, 2022), has been widely administered across Sub-Saharan Africa for onchocerciasis control (Mutono et al., 2024). The temporal correlation between mass ivermectin distribution and declining NS incidence has often been interpreted as evidence for a parasitic etiology. However, such an inference presumes that ivermectin acts exclusively through OV eradication and that removal of a single etiologic factor is sufficient to terminate disease risk. This assumption is inconsistent with both the drug's pleiotropic effects and the heterogeneous, nonlinear nature of NS susceptibility.

Elimination of OV may instead be understood as removal of one upstream stressor within a broader, multi-hit pathological cascade, thereby lessening a cumulative burden on vulnerable neuroimmune, metabolic, and epigenetic systems. Consistent with this interpretation, ivermectin exerts host-directed effects that extend well beyond antiparasitic activity, including modulation of GABA-A receptor signaling (Estrada-Mondragon and Lynch, 2015), regulation of purinergic pathways (Schneider et al., 2017), and influences on epigenetic states (Juarez et al., 2018). Ivermectin also alters gut microbial composition, including cyanobacterial populations (Ma et al., 2023), with downstream consequences for immune tone and metabolic signaling. Supporting a broader neuromodulatory role, ivermectin rescues seizure phenotypes in a *Xenopus laevis* tadpole model of Rett syndrome (Novak et al., 2022), implicating gut-brain-immune interactions that transcend pathogen clearance alone.

Importantly, ivermectin administration does not uniformly abolish NS risk, suggesting that OV removal is insufficient in subsets of hosts whose neurodevelopmental trajectories have already been destabilized. Moreover, ivermectin is associated with CNS adverse effects, including confusion, ataxia, seizures, and hypotension (Temple et al., 2021). This context-dependent duality, therapeutic modulation alongside potential neurotoxicity, highlights the complexity of its host-directed effects and cautions against simplistic causal inference based solely on epidemiological associations.

More broadly, this complexity illustrates a general principle relevant to NS pathophysiology: immune activation is not intrinsically pathological. Appropriately timed immune challenges in early life support microglial pruning and circuit maturation (Edridge et al., 2023), whereas chronic, excessive, or developmentally mistimed activation promotes maladaptive neurodevelopmental trajectories. Accordingly, therapeutic strategies for NS may benefit from immune recalibration rather than indiscriminate suppression, preserving essential host defense and repair mechanisms while limiting pathobiological activation.

In sum, effective interventions for NS are unlikely to be singular. Rather, durable benefits will likely require multipronged strategies targeting nodal vulnerabilities across the cascade identified in our model, including enhancement of proteostatic and autophagic clearance, stabilization of mitochondrial and metabolic function, preservation of excitatory-inhibitory balance, protection and remodeling of ECM integrity, and alleviation of pathological transcriptional repression through epigenetic or *MECP2*-modulating approaches. When coupled with early nutritional optimization and timely seizure management, such strategies may not only slow disease progression but also redirect the NS trajectory toward sustained resilience and functional recovery.

### 4.10 Toward a new paradigm

Earlier etiological models proposed OV infection as the primary cause of NS (Colebunders et al., 2017a; Van Cutsem et al., 2023). However, this view is increasingly untenable: OV remains endemic in geographical regions where NS has not been documented, postmortem analyses have failed to detect OV within the CNS (Pollanen et al., 2018), and a subset of recent-onset (< 1year) NS cases lack serological or clinical evidence of OV exposure (Edridge et al., 2023). These findings argue that OV is neither necessary nor sufficient for NS pathogenesis.

While ivermectin has proven essential in onchocerciasis control, its association with reduced NS incidence likely reflects broader host-directed immunomodulatory or neuroactive effects, rather than antiparasitic action alone (Section 4.9). Most critically, the evolving clinical and molecular profile of NS is better explained by a staged, systems-level collapse model encompassing: (i) prenatal disruption of immune and epigenetic programming, (ii) progressive intracellular collapse spanning proteostasis, cytoskeletal integrity, calcium homeostasis, mitochondrial function, and tau dynamics, and (iii) postnatal acceleration by environmental and metabolic stressors, culminating in transcriptional arrest, ECM degradation, and large-scale network desynchronization.

Within this framework, NS is best understood not as a geographically confined epilepsy of unknown origin, but as a complex neurodevelopmental disorder rooted in early-life immune reprogramming that destabilizes core regulatory systems. Central to this process is dysregulation of the *MECP2* axis, a hub integrating maternal adversity, metabolic fragility, oxidative stress, and neuroimmune signaling. Importantly, *MECP2* axis perturbation in NS may itself emerge downstream of immune imprinting, serving as both a mediator and amplifier of neural circuit instability.

NS thus occupies a unique pathological intersection: it features tau-associated proteostatic dysfunction akin to, but mechanistically distinct from, classical tauopathies such as AD (Kim et al., 2019), while concurrently mirroring the immune-metabolic fragility and synaptic instability central to neurodevelopmental conditions such as MDS and ASD (Liu et al., 2016; Nagarajan et al., 2006).

This paradigm shift, from narrow pathogen-centric models to broader systems-level developmental vulnerability, better explains NS's epidemiological heterogeneity, partial therapeutic responsiveness, and progressive clinical course. It also opens translational pathways toward preventive strategies, early detection, and interventions targeting immune-metabolic resilience during critical windows of brain development.

### 4.11 Limitations and future directions

#### 4.11.1 Limitations

This study has several limitations inherent to investigating a rare pediatric neurodegenerative condition in post-conflict, resource-limited settings. These constraints were addressed through analytical strategies designed to maximize interpretability and minimize bias.

##### 4.11.1.1 Cohort size

The modest sample size reflects the rarity of NS and the logistical constraints of pediatric CSF collection in affected regions. To mitigate reduced statistical power, we (i) employed edgeR, a framework well suited for small-sample proteomic analyses, and (ii) implemented a dual-consensus strategy, reporting only proteins significant across both the full cohort (*n* = 17) and a stringent QC-filtered subset (*n* = 12), thereby prioritizing robustness over sensitivity.

##### 4.11.1.2 Control selection and matching

Ethically appropriate, neurologically normal pediatric CSF controls are inherently unavailable in studies of this nature, and precise age- or sex-matching could not be achieved. Control samples were therefore obtained from children undergoing lumbar puncture for non-NS indications (e.g., meningitis, cerebral malaria). While such conditions may introduce inflammatory or neurovascular confounds, they lack progressive neurodegenerative pathology. Consequently, any shared proteomic signatures would be expected to bias findings conservatively, underestimating rather than overstating NS-specific effects. To further reduce noise, we applied a tiered analytical framework designed to prioritize robust, biologically relevant signals.

##### 4.11.1.3 Clinical metadata

Limited availability of detailed clinical and laboratory metadata (e.g., inflammatory indices, CSF cell counts) constrained our ability to fully adjust for sample-level confounding. As a partial safeguard, we assessed blood-associated CSF proteins (e.g., hemoglobin, albumin) and found no consistent evidence of contamination or pathological vascular leakage across samples.

##### 4.11.1.4 Indirect MECP2 inference

Consistent with its nuclear localization, MECP2 protein was not detected in CSF. Accordingly, inference of *MECP2*-axis involvement is indirect, based on convergent dysregulation of downstream pathways and reinforced by striking clinical parallels with *MECP2*-related neurodevelopmental syndromes. While this convergence supports biological plausibility, it does not constitute direct molecular confirmation.

##### 4.11.1.5 Environmental exposure and confounders

Direct epigenetic profiling and retrospective reconstruction of early-life exposures (e.g., MIA, biotoxins) were not possible due to the absence of maternal biospecimens and the inherent challenges of tracing transient, decades-old events. We therefore interpret the persistent proteomic alterations as plausible downstream imprints of early-life insults, while recognizing the need for prospective validation.

Chronic and overlapping environmental stressors prevalent in NS-prone regions (e.g., malnutrition, infectious burden, psychosocial stress) are likewise difficult to quantify retrospectively, yet likely contributed to both prenatal immune imprinting and postnatal vulnerability. While these exposures may underlie phenotypic heterogeneity, they do not contradict the central hypothesis of coordinated molecular collapse. Rather, their cumulative burden is integrated into the broader stress landscape and warrants prospective investigation, ideally through longitudinal human studies and controlled experimental models.

#### 4.11.2 . Future directions

To rigorously test and refine the proposed hypothesis, future research should integrate longitudinal, multi-omics approaches encompassing genomic, epigenomic, metabolomic, and environmental data while leveraging animal models exposed to both MIA and candidate postnatal stressors. Orthogonal validation of key proteomic signatures, particularly tau proteoforms and MECP2-associated targets, will be critical to establish mechanistic causality.

Ultimately, the path forward lies in preventive as much as therapeutic strategies: safeguarding maternal health, promoting early-life nutritional and metabolic resilience, minimizing cumulative toxic exposures, and recalibrating, rather than merely suppressing, immune responses. By intervening before vulnerable circuits cross thresholds of irreversible collapse, complex disorders like NS may be reclassified not as inevitable degenerative trajectories, but as preventable derailments of neurodevelopment.

## Data Availability

The original contributions presented in the study are publicly available. These data have been deposited to the ProteomeXchange Consortium via the PRIDE partner repository with the dataset identifier PXD068754.
