# Supplementary material for: The Nodding syndrome cerebrospinal fluid proteome: a lens into neurodevelopmental failure consistent with environmentally triggered MECP2 dysregulation?
Source: Front Mol Neurosci. 2026 Jan 29;19:1717920. doi: 10.3389/fnmol.2026.1717920 (PMC12894381; doi:10.3389/fnmol.2026.1717920)
Supplement: Supplementary file 1 [file Supplementary_file_1.docx]

**Supplemental File 1: Quantitative Proteomics Materials and Methods**

**Abundant protein depletion**

After sample collection, cerebrospinal fluids were stored in polypropylene centrifuge tubes at -80°C until digestion. To determine the protein concentration, a Pierce BCA protein assay (Thermo Fisher Scientific, Waltham, MA, Part# 23225) was performed on water-diluted cerebrospinal fluid samples. The average concentration of protein was 1.16 mg/mL.

Serum albumin was removed from the cerebrospinal fluid samples using a Human 14 Multiple Affinity Removal Spin Cartridge (Agilent, Santa Clara, CA, Part#5188-6560). To prepare the samples for albumin depletion, a 1000 µl aliquot of cerebrospinal fluid was transferred to a 1.5mL Protein LoBind centrifuge tube (VWR, Radnor, PA, Part# 80077-232) and centrifuged at 14,000 xg for 10 minutes. The supernatant was transferred to a 0.22 µm Ultrafree-MC PVDF spin filter (Millipore, Burlington, MA, Part# UFC30GVNB). The samples were centrifuged until all the cerebrospinal fluid had passed through the filter. The 0.22 µm filtrate was transferred to an Amicon Ultracel-3K 3,000 NMWL centrifugal filter (Millipore, Burlington, MA, Part# UFC500396) and centrifuged at 14,000 xg until the retentate volume was approximately 80 µL. Column Buffer A buffer exchanged retentate was collected by inverting the 3,000 Dalton centrifugal filter into a clean 2.0 mL centrifuge tube and centrifuged at 3,000 xg for 3 minutes. The volume was measured, and additional Depletion Column Buffer A was added to bring the final volume to 200 µl.

The depletion cartridge was equilibrated with Depletion Column Buffer A and 200 µl of sample was added to the top of the cartridge. The cartridge was centrifuged at 100 xg for 1.5 minutes and the flow through was collected. To the top of the depletion cartridge, 400 µl of Depletion Column Buffer A was added and the cartridge centrifuged at 100 xg for 1.5 minutes and the flow through was collected. An additional 400 µl of Depletion Column Buffer A was added to the top of the cartridge and centrifuged at 100 xg for 1.5 minutes and the flow through was collected. The depletion spin cartridge was cleaned by eluting the bound human serum albumin with 400 µl of Depletion Column Buffer B (Agilent, Santa Clara, CA, Part# 5185-5988). The three flow-through fractions were combined and concentrated in a new 3,000 NMWL Amicon centrifugal spin filter. Sample was added in 400 µl increments to the centrifugal filter which was centrifuged at 14,000 xg for 10 minutes between additions. The now concentrated sample retentate was collected by inverting the 3,000 Dalton centrifugal filter into a clean 2.0 mL centrifuge tube and centrifuged at 3,000 xg for 3 minutes. The volume was measured, and a Pierce BCA protein assay (Thermo Fisher Scientific, Waltham, MA) was performed to determine the post-depletion protein recovery. The average amount of top 14 depleted protein was 31.9 µg with an average recovery of 7.9%.

**Digestion**

Twenty µg of proteins per sample were digested using an EasyPep Mini Digestion kit (Thermo Fisher Scientific, Waltham, MA). The concentrated sample retentate was dried using a Speedvac centrifugal vacuum. The resulting pellet was resuspended in 100 µl of EasyPep Mini lysis buffer and incubated at 50°C for 15 minutes. The samples were reduced and the cysteines alkylated by adding 50 µl each of the EasyPep Mini Reduction Solution and the EasyPep Mini Alkylation Solution and incubating the resultant mixture at 95°C for 10 minutes. Trypsin/Lys-C was added, and the samples were incubated with shaking at 37°C for 3 hours. The digestion was halted with 50 µl of the Digestion Stop Solution and the samples were gently mixed for 10 seconds.

Digested samples were cleaned up using the Peptide Clean-up column included in the EasyPep Mini kit. The digest solution was transferred to a dry Clean-up column and centrifuged at 1,500 xg for 2 minutes. The column was washed once with 300 µl of Wash Solution A and twice with 300 µl of Wash Solution B with the buffer being removed by centrifugation at 1,500 xg for 2 minutes in between washes. Peptides were eluted from the Peptide Clean-up column using 300 µl of the Elution Solution followed by centrifugation at 1,500 xg for 2 minutes. The eluent was dried using Speedvac centrifugal vacuum and the peptide samples reconstituted in 100ul of 0.1% formic acid and peptide concentrations determined using a Pierce Quantitative Colorimetric Peptide Assay (Thermo Fisher Scientific, Cat # 23275). For the TMTpro labeling step, 6.8 µg of the peptide from each cerebrospinal fluid sample were dried by vacuum centrifugation. Dried peptides were reconstituted by adding 100 µL of 100mM Tetraethylammonium bromide (TEAB) and shaking at 37°C for 15 minutes.

**TMTpro labeling**

Two tandem mass tag (TMTpro) 18-plex reagent kits (Thermo Fisher Scientific, Cat # 90309) were used to label the digested cerebrospinal fluid peptide samples and pooled standards. Twelve µl of anhydrous acetonitrile was added to 100 µg portions of TMTpro 18-plex reagents, the peptide samples in 20 µl of 100mM TEAB were immediately transferred into the dissolved TMTpro reagents, and labeling was performed by shaking at room temp for 1 hr. After the incubation, 2 µl of each labeled peptide was combined, and 2 µl of 5% hydroxylamine was added. Samples were incubated at room temp for 15 min, then dried by vacuum centrifugation. The remaining 30 µl of each labeled sample was frozen at -80°C without hydroxylamine addition, in case relabeling was required.

The 2 µl of each combined TMTpro labeled sample was then dissolved in 20 µl of 5% formic acid and 2 µg of peptides analyzed by a single 140-min LC-MS/MS method using an Orbitrap Eclipse Mass Spectrometer, as described below. This LC run was performed to check labeling efficiency (typically >90%) and volumetrically adjust each sample to provide equal total reporter ion intensities for each labeled sample in the final combined sample for the 2D-LC/MS analysis. The remaining 30 µl portion of each TMTpro labeled sample was then thawed and aliquots were removed and adjusted by the calculated normalization factors that would produce 32 µg of total peptides from all 18 samples with equal total reporter ion intensities. To quench the labeling reaction, 5% hydroxylamine was added to bring the total hydroxylamine concentration to 0.5% followed by incubation for 15 min at room temperature. The remaining labeled peptides not used for the 2D-LC/MS were stored at -80°C in case a second run was needed.

**Two-dimensional liquid chromatography/mass spectrometry (2D-LC/MS) analysis**

The multiplexed samples were dissolved in 10 mM ammonium formate, pH = 9 buffer and injected onto a NanoEase 5 µm XBridge BEH130 C18 300 µm x 50 mm column (Waters Corporation, Milford, MA) at 3 µl/min in a mobile phase containing 10 mM ammonium formate (pH 9). Peptides from human cerebrospinal fluid were eluted by sequential injection of 20 µl volumes of 17, 20, 21, 22, 23, 24, 25, 26, 27, 28, 29, 30, 31, 32, 33, 34, 35, 37, 40, 45, 50, 90% ACN (22 fractions) for analysis using a Dionex NCS-3500RS UltiMate RSLCnano UPLC (Thermo Fisher Scientific).

Eluted peptides were diluted at a 3-way union with mobile phase containing 0.1% formic acid at a 24 µl/min flow rate and delivered to an Acclaim PepMap 100 µm x 2 cm NanoViper C18, 5 µm trap (Thermo Fisher Scientific) on a switching valve. After 10 min of loading, the trap column was switched in-line to a PepMap RSLC C18, 2 µm, 75 µm x 25 cm EasySpray column (Thermo Fisher Scientific). TMTpro 18-plex labeled peptides from cerebrospinal fluid were then separated at low pH in the second dimension using a 7.5–30% ACN gradient over 100 min in the mobile phase containing 0.1% formic acid at a 300 nL/min flow rate.

Tandem mass spectrometry data were collected using an Orbitrap Eclipse Tribrid instrument (Thermo Fisher Scientific, San Jose, CA) configured with an EasySpray NanoSource. Survey scans were performed in the Orbitrap mass analyzer at resolution = 120,000, with internal mass calibration enabled, and data-dependent MS2 scans using dynamic exclusion performed in the linear ion trap using collision-induced dissociation. Reporter ion detection was performed in the Orbitrap mass analyzer using MS3 scans following synchronous precursor isolation of the top 10 ions in the linear ion trap, and higher-energy collisional dissociation in the ion-routing multipole. Full instrument parameters are in the table below.

**Orbitrap Eclipse Settings:** Instrument control software version 3.4.3072.18

| General Settings |  |  |
| --- | --- | --- |
|  | Method duration | 140 min |
|  | Ion source type | NSI |
|  | Spray voltage positive ion | 2000 V |
|  | Ion transfer tube temperature | 300 C |
|  | Internal mass calibration | RunStartEasyIC |
| MS1 Scans |  |  |
|  | Detector | Orbitrap |
|  | MS1 resolution | 120,000 |
|  | Scan range | 375 to 1500 m/z |
|  | Maximum inject time | 246 ms |
|  | AGC target | 400,000 (100%) |
|  | Microscans | 1 |
|  | MIPS mode | Peptide |
|  | Minimum intensity | 5000 |
|  | Charge states | 2 to 5 |
|  | Include undetermined charge states | FALSE |
| Dynamic exclusion settings |  |  |
|  | Duration | 45 s |
|  | Mass tolerance low | 10 ppm |
|  | Mass tolerance high | 10 ppm |
|  | Exclude isotopes | TRUE |
|  | Single charge state per precursor | TRUE |
| MS2 Scans |  |  |
|  | Detector | Linear ion trap |
|  | Quadrupole isolation | 1 m/z, no offset |
|  | Fragmentation | CID |
|  | Normalized collision energy | 35 % |
|  | Activation | 10 ms with Q=0.25 |
|  | Scan rate | Rapid |
|  | Maximum inject time | 75 ms |
|  | AGC target | 20,000 |
|  | Scan range | Auto mode |
|  | Microscans | 1 |
| MS3 scans |  |  |
|  | Detector | Orbitrap |
|  | MS2 isolation window | 2 |
|  | Number of notches | 10 |
|  | Fragmentation | HCD |
|  | Normalized collision energy | 55 % |
|  | Resolution | 50,000 |
|  | Scan range | 100 to 150 |
|  | Maximum inject time | 86 ms |
|  | AGC target | 100,000 (200%) |
|  | Microscans | 1 |

**Dionex NCS-3500RS UltiMate Settings:** Software Version: Thermo SII 1.5.0.10747

| Buffer Info |  |  |
| --- | --- | --- |
|  | 2D Loading Mobile Phase | 10 mM Ammonium Formate, pH 9 |
|  | 1D Mobile Phase A | 0.1% Formic Acid in Water |
|  | 1D Mobile Phase B | 0.1% Formic Acid in Acetonitrile |
| 2D HPLC |  |  |
|  | 2DRPRP Trap Column | Waters NanoEase XBridge BEH130 C18 300 µm x 50 mm column, 5 µm |
|  | Flow Rate | 24 µL/min |
|  | Loading Time | 10 minutes |
| 1D HPLC |  |  |
|  | Trap Column | Thermo Acclaim PepMap C18 100 µm x 2 cm NanoViper, 5 µm |
|  | Analytical Column | Thermo PepMap RSLC C18, 75 µm x 25 cm EasySpray, 2 µm |
|  | Flow Rate | 300 nL/min |
|  | Run Time | 140 min |
|  | Starting Mobile Phase | 2% Mobile Phase B |
| 1D Gradient Profile |  |  |
|  | *Time* | *Mobile Phase B Composition* |
|  | 0.0-10.0 min | 2% |
|  | 10.0-10.1 min | 5% |
|  | 10.1-110.0 min | 25% |
|  | 110.0-114.0 min | 95% |
|  | 114.0-119.0 min | 95% |
|  | 119.0-120.0 min | 2% |
|  | 120.0-140.0 min | 2% |

**Data analysis**

Proteome UP000005640 (Homo Sapiens, taxon ID 9606) canonical FASTA sequences (20.650 proteins) were downloaded January 2025 from www.UniProt.org. Common contaminants (175 sequences) were added, and sequence-reversed entries were concatenated for a final protein FASTA file of 41,650 sequences.

The 22 binary instrument files were processed with the PAW pipeline (1). Binary files were converted to text files using MSConvert (2). Python scripts extracted TMTpro reporter ion peak heights and fragment ion spectra in MS2 format (3). The Comet search engine (version 2016.03) (4) was used: 1.25 Da monoisotopic peptide mass tolerance, 1.0005 Da monoisotopic fragment ion tolerance, semi tryptic cleavage with up to three missed cleavages, variable oxidation of methionine residues, static alkylation of cysteines, and static modifications for TMTpro labels (at peptide N-termini and at lysine residues).

Top-scoring peptide spectrum matches (PSMs) were filtered to a 1% false discovery rate (FDR) using interactive delta-mass and conditional Peptide-prophet-like linear discriminant function (5) scores. Incorrect delta-mass and score histogram distributions were estimated using the target/decoy method (6). The filtered PSMs were assembled into protein lists using basic and extended parsimony principles and required two distinct peptides per protein. The final list of identified proteins, protein groups, and protein families were used to define unique and shared peptides for quantitative use. Total (summed) reporter ion intensities were computed from the PSMs associated with all unique peptides for each protein.

The protein intensity values for each biological sample in each biological condition were compared for differential protein expression using the Bioconductor package edgeR (7) within Jupyter notebooks. Result tables contained typical proteomics summaries, reporter ion intensities, and statistical testing results. Additional annotations from [www.UniProt.org](http://www.UniProt.org) were added (<https://github.com/pwilmart/annotations>).

The mass spectrometry proteomics data have been deposited to the ProteomeXchange Consortium (<http://proteomecentral.proteomexchange.org>) via the PRIDE partner repository (8) with the dataset identifier PXD068754.

**References**

1. Wilmarth PA, Riviere MA, David LL. Techniques for accurate protein identification in shotgun proteomic studies of human, mouse, bovine, and chicken lenses. J Ocul Biol Dis Infor (2009) 2:223-234. doi: 10.1007/s12177-009-9042-6
2. Chambers MC, Maclean B, Burke R, Amodei D, Ruderman DL, Neumann S, et al. A cross-platform toolkit for mass spectrometry and proteomics. Nat Biotechnol (2012) 30:918-920. doi: 10.1038/nbt.2377
3. McDonald WH, Tabb DL, Sadygov RG, MacCoss MJ, Venable J, Graumann J, et al. MS1, MS2, and SQT—three unified, compact, and easily parsed file formats for the storage of shotgun proteomic spectra and identifications. Rapid Commun Mass Spectrom (2004) 18:2162-2168. doi: 10.1002/rcm.1603
4. Eng JK, Jahan TA and Hoopmann MR. Comet: an open‐source MS/MS sequence database search tool. Proteomics (2013) 13:22-24. doi: 10.1002/pmic.201200439
5. Keller A, Nesvizhskii AI, Kolker E, Aebersold R. Empirical statistical model to estimate the accuracy of peptide identifications made by MS/MS and database search. Anal Chem (2002) 74:5383-5392. doi: 10.1021/ac025747h
6. Elias JE, Gygi SP. Target-decoy search strategy for increased confidence in large-scale protein identifications by mass spectrometry. Nat Methods (2007) 4:207-214. doi: 10.1038/nmeth1019
7. Robinson MD, McCarthy DJ, Smyth GK. edgeR: a Bioconductor package for differential expression analysis of digital gene expression data. Bioinformatics (2010) 26:139-140. doi: 10.1093/bioinformatics/btp616
8. Perez-Riverol Y, Bai J, Bandla C, García-Seisdedos D, Hewapathirana S, Kamatchinathan S, et al. The PRIDE database resources in 2022: a hub for mass spectrometry-based proteomics evidences. Nucleic Acids Res (2022) 50:D543-D552. doi: 10.1093/nar/gkab1038
