# Supplementary material for: The Nodding syndrome cerebrospinal fluid proteome: a lens into neurodevelopmental failure consistent with environmentally triggered MECP2 dysregulation?
Source: Front Mol Neurosci. 2026 Jan 29;19:1717920. doi: 10.3389/fnmol.2026.1717920 (PMC12894381; doi:10.3389/fnmol.2026.1717920)
Supplement: Supplementary file 3 [file Table_1.docx]

**Supplemental Table 1.** **Genes with corresponding protein names and functions.**

The table lists standardized gene symbols, official protein nomenclature, and a concise description of the primary biological function(s) of each protein.

| Gene Symbol | Protein Name | Concise Protein Function |
| --- | --- | --- |
| *ACLY* | ATP citrate lyase | Catalyzes cytosolic acetyl-CoA synthesis from citrate; links carbohydrate metabolism to fatty-acid and cholesterol biosynthesis. |
| *ACOX1* | Acyl-CoA oxidase 1 | Peroxisomal enzyme catalyzing the first step of very-long-chain fatty-acid β-oxidation. |
| *ACTR2* | Actin-related protein 2 (ARP2) | ARP2/3 complex subunit; nucleates branched actin filaments and organizes cortical actin networks. |
| *ACTR3* | Actin-related protein 3 (ARP3) | ARP2/3 complex subunit; cooperates with ACTR2 to initiate branched actin polymerization. |
| *ADH5 (aka ADH3)* | Alcohol dehydrogenase 5 (class III; GSNOR) | Class III alcohol dehydrogenase (S-nitrosoglutathione reductase); metabolizes formaldehyde and nitrosothiols, contributing to redox homeostasis. |
| *AEBP1* | Adipocyte enhancer-binding protein 1 | Zinc-finger transcriptional regulator implicated in extracellular matrix organization and inflammatory signalling. |
| *AHCY* | S-adenosylhomocysteine hydrolase (SAHH) | Hydrolyzes S-adenosylhomocysteine to homocysteine and adenosine; controls cellular methylation potential. |
| *AKR1A1* | Aldo-keto reductase family 1 member A1 | NADPH-dependent oxidoreductase that reduces aldehydes/ketones; involved in detoxification. |
| *AKR1B1* | Aldo-keto reductase family 1 member B1 (aldose reductase) | Reduces glucose-derived aldehydes and other carbonyls; implicated in osmotic/diabetic stress responses. |
| *ALDH1A1* | Aldehyde dehydrogenase 1 family member A1 | Oxidizes retinaldehyde to retinoic acid; detoxifies reactive aldehydes. |
| *ALDOB* | Fructose-bisphosphate aldolase B | Catalyzes cleavage of fructose-1,6-bisphosphate in glycolysis/gluconeogenesis; fructose metabolism in liver. |
| *ALDOC* | Fructose-bisphosphate aldolase C | Brain/muscle isoform of aldolase; functions in glycolytic flux in neural tissue. |
| *AMICA1* | Junctional adhesion molecule-like protein (JAML) | Mediates leukocyte adhesion and transmigration in inflammatory responses. |
| *ANXA5* | Annexin A5 | Ca²⁺-dependent phospholipid-binding protein; marks apoptotic cells and has anticoagulant activity. |
| *APEX1* | Apurinic/apyrimidinic endonuclease 1 (APE1/Ref-1) | Central base excision-repair endonuclease and redox modulator of transcription factors. |
| *APP* | Amyloid precursor protein (APP) | Membrane glycoprotein involved in synaptogenesis and axonal growth; precursor of amyloid-β peptides. |
| *ASAH1* | Acid ceramidase (ASAH1) | Lysosomal enzyme converting ceramide to sphingosine + fatty acid; regulates sphingolipid signalling and apoptosis. |
| *ASTN2* | Astrotactin-2 | Cell-adhesion protein involved in neuronal migration, synaptic function and receptor trafficking. |
| *ATP6V1 / ATP6V0 (subunits)* | Vacuolar type H+-ATPase or (V)-ATPase (subunits) | Proton pump subunits that acidify endosomes/lysosomes; essential for proteolysis, trafficking and autophagy. |
| *BDNF* | Brain-derived neurotrophic factor | Neurotrophin that supports neuronal survival, differentiation and synaptic plasticity. |
| *BMPs (family)* | Bone morphogenetic proteins (TGF-β family) | Secreted morphogens that regulate neuronal differentiation, patterning and tissue morphogenesis. |
| *BPGM* | Bisphosphoglycerate mutase | Produces 2,3-BPG in erythrocytes, regulating hemoglobin O₂ affinity. |
| *BRINP1* | BMP/retinoic acid-inducible neural-specific protein 1 | Modulates neurogenesis and neuronal differentiation during development. |
| *C1QTNF4* | C1q and TNF-related protein 4 (CTRP4) | Secreted adipokine-like protein implicated in metabolic and inflammatory signalling. |
| *C2* | Complement component 2 | Classical pathway complement component required for C3 convertase formation. |
| *C4A* | Complement component 4A | Classical/lectin pathway component; C4 cleavage into C4a and C4b: C4a acts as an anaphylatoxin and C4b functions as an opsonin in immune complex clearance. |
| *C4BPA / C4BPB* | C4b-binding protein α and β chain | Soluble regulator that binds C4b and acts as a cofactor for factor I to limit complement activation. |
| *C5* | Complement component 5 | Cleaved to C5a (potent anaphylatoxin) and C5b (initiator of membrane attack complex -MAC- assembly); mediates inflammation and host defense. |
| *C6* | Complement component 6 | Terminal complement component that participates in membrane attack complex (MAC) formation. |
| *C8A / C8B* | Complement component 8 subunits | MAC subunits required for pore formation and cell lysis. |
| *CALB1* | Calbindin | Calcium-binding protein that buffers intracellular Ca²⁺ and shapes neuronal calcium signaling and excitability. |
| *CALM1* | Calmodulin 1 | Calcium sensor protein that binds Ca²⁺ and modulates diverse targets involved in synaptic transmission, plasticity, and signal transduction. |
| *CAMK2 (e.g., CAMK2A/B)* | Calcium/calmodulin-dependent protein kinase II | Ser/Thr kinase critical for Ca²⁺-dependent signalling in synaptic plasticity, learning and memory. |
| *CASP3* | Caspase-3 | Executioner caspase in apoptosis; cleaves multiple substrates. |
| *CAT* | Catalase | Peroxisomal enzyme converting H₂O₂ to H₂O and O₂; antioxidant defense. |
| *CEL* | Carboxyl ester lipase (bile salt-activated lipase) | Pancreatic enzyme that hydrolyzes dietary esters and triglycerides. |
| *CFB* | Complement factor B | Alternative pathway component; Bb fragment forms the alternative pathway C3 convertase. |
| *CFI* | Complement factor I | Serine protease that cleaves/inactivates C3b and C4b to downregulate complement. |
| *CFP* | Properdin | Positive regulator of the alternative complement pathway; stabilizes C3/C5 convertases. |
| *CD200* | CD200 (OX-2 membrane glycoprotein) | Immune inhibitory ligand that suppresses macrophage/microglial activation. |
| *CFL1* | Cofilin-1 | Actin-binding protein that severs and depolymerizes actin filaments; controls cytoskeletal dynamics. |
| *CLIC1* | Chloride intracellular channel 1 | Intracellular chloride channel implicated in ion homeostasis and redox responses. |
| *COX6B1* | Cytochrome c oxidase subunit 6B1 | Structural subunit of mitochondrial complex IV; required for electron transport. |
| *CTSD* | Cathepsin D | Lysosomal aspartic protease involved in protein turnover and apoptosis. |
| *CTSL* | Cathepsin L | Lysosomal cysteine protease; roles in protein degradation, antigen processing and ECM remodelling. |
| *DDX39B* | DEAD-box helicase 39B | RNA helicase in the THO/TREX complex; involved in mRNA processing and nuclear export. |
| *DYNC1LI2* | Dynein cytoplasmic 1 light intermediate chain 2 | Dynein complex subunit mediating retrograde transport and organelle motility. |
| *ECI1* | Enoyl-CoA delta isomerase 1 | Mitochondrial enzyme in β-oxidation of unsaturated fatty acids. |
| *EEF2* | Eukaryotic translation elongation factor 2 | Catalyzes ribosomal translocation during protein synthesis. |
| *EIF4G1* | Eukaryotic translation initiation factor 4 gamma 1 | Scaffolding subunit of eIF4F complex required for cap-dependent translation initiation. |
| *EIF5A (EIF5A1)* | Eukaryotic translation initiation factor 5A 1 | Translation factor requiring hypusination; involved in translation elongation and cell growth. |
| *ENO1* | Alpha-enolase | Glycolytic enzyme (2-phosphoglycerate → phosphoenolpyruvate); also moonlights as a plasminogen receptor and stress-response protein. |
| *F5* | Coagulation factor V | Cofactor in prothrombinase complex; accelerates thrombin formation. |
| *F9* | Coagulation factor IX | Vitamin K-dependent serine protease in the intrinsic coagulation pathway. |
| *F11* | Coagulation factor XI | Serine protease activating factor IX in intrinsic coagulation. |
| *F13A1* | Coagulation factor XIII A chain | Transglutaminase that crosslinks fibrin to stabilize clots. |
| *FCER2* | Low-affinity IgE receptor (CD23) | Regulates IgE-dependent responses and B-cell differentiation. |
| *FCN3* | Ficolin-3 (H-ficolin) | Lectin pathway pattern recognition molecule that binds carbohydrates on microbes. |
| *FGG* | Fibrinogen γ chain | Component of fibrinogen; essential for fibrin clot formation. |
| *FKBP1A* | FK506-binding protein 1A (FKBP12) | Peptidyl-prolyl isomerase and immunophilin that modulates Ca²⁺ channels and signaling. |
| *FLNA* | Filamin A | Actin crosslinking scaffold that organizes signaling complexes and membrane proteins. |
| *GABBR1* | GABA type B receptor subunit 1 | G-protein coupled receptor mediating slow inhibitory neurotransmission. |
| *GALC* | Galactocerebrosidase | Lysosomal enzyme hydrolyzing galactosylceramide; essential for myelin lipid turnover. |
| *GPC6* | Glypican-6 | Heparan-sulfate proteoglycan modulating growth-factor signalling (e.g., Wnt). |
| *GPI* | Glucose-6-phosphate isomerase | Glycolytic enzyme; extracellularly acts as autocrine motility factor. |
| *HDGF* | Hepatoma-derived growth factor | Mitogenic growth factor implicated in proliferation and angiogenesis. |
| *HLA-A / HLA-B / HLA-C* | MHC class I heavy chains | Present endogenous antigenic peptides to CD8⁺ T cells; immune surveilance |
| *HLA-DP / HLA-DQ / HLA-DR* | MHC class II molecules | Present exogenous peptides to CD4⁺ T cells; adaptive immunity |
| *HNRNP (family)* | Heterogeneous nuclear ribonucleoproteins (hnRNPs) | Pre-mRNA processing, splicing, transport and mRNA stability regulators. |
| *HMGA1* | High mobility group AT-hook 1 | Chromatin architectural protein modulating transcriptional programs. |
| *HMGB1* | High mobility group box 1 | Nuclear DNA-binding protein that acts extracellularly as an alarmin. |
| *HMOX1* | Heme oxygenase 1 (HO1) | Catalyzes heme breakdown to biliverdin, CO and iron; cytoprotective antioxidant response. |
| *HSPA8* | Heat shock cognate 71 kDa protein (HSC70) | Constitutive HSP70 family chaperone; protein folding, quality control, autophagy. |
| *HSP90AA1* | Heat shock protein 90 a  (HSP90a) | Molecular chaperone stabilizing client proteins and regulating signalling. |
| *IDH1* | Isocitrate dehydrogenase 1 (cytosolic) | NADP⁺-dependent enzyme producing NADPH; links metabolism and redox homeostasis. |
| *IGHM* | Immunoglobulin heavy constant m | Encodes μ heavy chain of IgM antibodies; primary humoral response. |
| *ILF2* | Interleukin enhancer-binding factor 2 (NF45) | RNA-binding protein involved in transcriptional regulation and RNA processing. |
| *IRAK1* | Interleukin-1 receptor-associated kinase 1 | Kinase mediating TLR/IL-1R signalling in innate immune responses. |
| *ITGB2* | Integrin β2 (CD18) | β subunit of leukocyte integrins (LFA-1, Mac-1); mediates leukocyte adhesion and migration. |
| *ITIH4* | Inter-α-trypsin inhibitor heavy chain H4 | Acute-phase ECM-associated protein involved in protease inhibition and matrix stabilization. |
| *KLK family* | Kallikrein-related peptidases | Secreted serine proteases processing peptides and regulating diverse physiological processes. |
| *KREMEN1* | Kremen protein 1 | High-affinity receptor for DKK proteins that mediates Wnt receptor endocytosis and pathway inhibition. |
| *LBP* | Lipopolysaccharide-binding protein | Binds and transfers bacterial LPS to CD14/TLR4 to initiate innate signalling. |
| *LTA4H* | Leukotriene A4 hydrolase | Generates pro-inflammatory leukotriene B4 and degrades PGP peptide; dual enzymatic roles. |
| *MAP1LC3B* | Microtubule-associated protein 1 light chain 3b (LC3B) | Autophagosome membrane component; canonical autophagy marker. |
| *MAP2* | Microtubule-associated protein 2 | Neuronal microtubule stabilizer that supports dendritic architecture. |
| *MMP3* | Matrix metalloproteinase-3 (stromelysin-1) | ECM protease involved in matrix remodelling and activation of other MMPs. |
| *MMP8* | Matrix metalloproteinase-8 (neutrophil collagenase) | Collagenase released by neutrophils; participates in inflammatory ECM degradation. |
| *MMP9* | Matrix metalloproteinase-9 (gelatinase B) | Degrades ECM components and modulates BBB permeability and inflammation. |
| *MNDA* | Myeloid cell nuclear differentiation antigen | Nuclear protein expressed in myeloid cells; implicated in innate immune transcriptional programs. |
| *MPO* | Myeloperoxidase | Neutrophil heme enzyme producing hypochlorous acid for microbicidal activity and oxidative stress. |
| *NGFR* | Nerve growth factor receptor (p75NTR) | Low-affinity neurotrophin receptor regulating survival, apoptosis and neurite outgrowth. |
| *NLGN1* | Neuroligin-1 | Postsynaptic cell-adhesion molecule that organizes excitatory synapses via neurexin binding. |
| *NLGN3* | Neuroligin-3 | Synaptic cell-adhesion protein involved in synapse formation and function. |
| *NPM1* | Nucleophosmin | Nucleolar chaperone involved in ribosome biogenesis, DNA repair and genome stability. |
| *NPC2* | Niemann-Pick disease type C2 protein | Lysosomal sterol-binding protein required for intracellular cholesterol trafficking. |
| NTRK2 | Neurotrophic receptor tyrosine kinase 2 (TrkB) | Receptor tyrosine kinase mediating BDNF/NT-4 signaling to regulate neuronal survival, differentiation, and synaptic plasticity. |
| *ORM1* | Orosomucoid 1 (α-1-acid glycoprotein 1) | Acute-phase plasma glycoprotein that modulates immune responses and drug binding. |
| *ORM2* | Orosomucoid 2 (α-1-acid glycoprotein 2) | Acute-phase glycoprotein with functions overlapping ORM1. |
| *PAICS* | Phosphoribosylaminoimidazole succinocarboxamide synthetase | Enzyme in de novo purine biosynthesis catalyzing successive steps toward AMP synthesis. |
| *PARK7* | Parkinsonism-associated deglycase (DJ-1) | Oxidative-stress sensor and chaperone implicated in mitochondrial protection and Parkinson’s disease. |
| *PF4* | Platelet factor 4 (CXCL4) | Platelet-derived chemokine that modulates coagulation and innate immune cell recruitment. |
| *PFN1* | Profilin-1 | Actin-binding protein that promotes actin polymerization and links signaling to cytoskeleton. |
| *PGAM1* | Phosphoglycerate mutase 1 | Glycolytic enzyme interconverting 3-PG and 2-PG; important for energy metabolism. |
| *PGD* | 6-Phosphogluconate dehydrogenase | Oxidative branch enzyme of pentose phosphate pathway generating NADPH. |
| *PGK1* | Phosphoglycerate kinase 1 | Glycolytic ATP-generating enzyme (1,3-BPG → 3-PG). |
| *PKM* | Pyruvate kinase M (M1/M2 isoforms) | Catalyzes PEP → pyruvate in glycolysis and regulates metabolic flux. |
| *PLG* | Plasminogen | Zymogen of plasmin; mediates fibrinolysis and ECM degradation. |
| *PNP* | Purine nucleoside phosphorylase | Catalyzes purine nucleoside catabolism; important for nucleotide salvage. |
| *PPIA* | Peptidyl-prolyl cis-trans isomerase A (cyclophilin A) | Prolyl isomerase chaperone participating in protein folding and inflammatory signalling. |
| *PSME1* | Proteasome activator complex subunit 1 (PA28α/REGα) | Proteasome regulator that modulates antigen processing. |
| *PTBP1* | Polypyrimidine tract binding protein 1 (PTB1) | RNA-binding splicing regulator that controls alternative splicing and mRNA metabolism. |
| *PTGES3* | Prostaglandin E synthase 3 (p23) | HSP90 co-chaperone with roles in prostaglandin biosynthesis and protein folding. |
| *PYCARD* | ASC (apoptosis-associated speck-like protein containing a CARD) | Inflammasome adaptor essential for caspase-1 activation and pyroptosis. |
| *PYGL* | Glycogen phosphorylase, liver form | Catalyzes glycogenolysis to release glucose-1-phosphate; key for hepatic glucose supply. |
| *RAB1B* | Ras-related protein Rab-1B | Small GTPase controlling ER→Golgi vesicle trafficking. |
| *RAB11A* | Ras-related protein Rab-11A | Small GTPase regulating recycling endosome trafficking and return of membrane proteins to the plasma membrane. |
| *RAB5C* | Ras-related protein Rab-5C | Small GTPase controlling early endosome formation, endocytic vesicle fusion, and receptor internalization. |
| *RABGDIB* | Rab GDP dissociation inhibitor beta (GDIβ) | Regulator of Rab GTPases that controls Rab cycling between membranes and cytosol by inhibiting GDP dissociation. |
| *RBBP4* | Retinoblastoma-binding protein 4 | Chromatin remodeler and histone-binding subunit of NuRD and related complexes. |
| *RELN* | Reelin | Secreted ECM glycoprotein guiding neuronal migration and synaptic plasticity. |
| *RHO family (e.g., RHOA)* | Rho GTPases | Master regulators of actin cytoskeleton, cell adhesion and migration. |
| *RPA1* | Replication protein A 70 kDa subunit | ssDNA-binding factor essential for replication and DNA repair. |
| *ROR1* | Receptor tyrosine kinase-like orphan receptor 1 | Developmental receptor that modulates Wnt signalling; aberrant expression in cancer. |
| *S100A8* | S100 calcium-binding protein A8 (MRP8) | Inflammatory alarmin forming heterodimer with S100A9 to regulate neutrophil function. |
| *S100A9* | S100 calcium-binding protein A9 (MRP14) | Partner of S100A8; modulates ROS production and inflammatory signalling. |
| *SAA1 / SAA2* | Serum amyloid A proteins | Acute-phase apolipoproteins involved in inflammation and lipid transport. |
| *SARAF* | Store-operated calcium entry-associated regulatory factor | Negative regulator of store-operated Ca²⁺ entry; maintains Ca²⁺ homeostasis. |
| *CD163* | Scavenger receptor cysteine-rich type 1 protein M130 | Hemoglobin-haptoglobin scavenger receptor on macrophages; soluble form (sCD163) is an anti-inflammatory marker. |
| *SEMA6A* | Semaphorin-6A | Transmembrane guidance cue for axon pathfinding and neuronal connectivity. |
| *SERPINA3* | Alpha-1-antichymotrypsin | Acute-phase serine protease inhibitor implicated in inflammation and neurodegeneration. |
| *SFTPD* | Surfactant protein D | Innate immune collectin in lung that mediates opsonization and clearance of pathogens. |
| *SLC8A1* | Sodium/calcium exchanger 1 (NCX1) | Electrogenic exchanger exporting Ca²⁺ in exchange for Na⁺; crucial for Ca²⁺ homeostasis. |
| *SIL1* | SIL1 nucleotide exchange factor (BAP) | ER co-chaperone required for BiP nucleotide exchange and protein folding. |
| *SNCA* | Alpha-synuclein | Presynaptic protein regulating vesicle dynamics; aggregation implicated in synucleinopathies. |
| *SNRNPs (family)* | Small nuclear ribonucleoproteins (snRNPs) | Core spliceosomal components required for pre-mRNA splicing. |
| *SORT1* | Sortilin | **Acts as a co-receptor with p75NTR**to mediate **neuronal apoptosis**, especially in response to **pro-neurotrophins** like proNGF and proBDNF. |
| *SRSF1* | Serine/arginine-rich splicing factor 1 | Regulator of alternative splicing and mRNA export. |
| *SRSF2* | Serine/arginine-rich splicing factor 2 | Regulator of splice site selection and pre-mRNA processing. |
| *SRRM2* | Serine/arginine repetitive matrix protein 2 (SRm300) | Spliceosome-associated factor organizing nuclear speckles and splicing complexes. |
| *TALDO1* | Transaldolase 1 | Non-oxidative pentose phosphate pathway enzyme involved in sugar interconversions. |
| *TCEAL6* | Transcription elongation factor A-like 6 | Putative regulator of transcription elongation and gene expression. |
| *TPT1* | Translationally-controlled tumor protein (TCTP) | Multifunctional protein implicated in apoptosis, protein synthesis and cell proliferation. |
| *TLN1* | Talin-1 | Cytoskeletal adaptor linking integrins to actin to mediate adhesion and mechanotransduction. |
| *TNFSF12* | Tumor necrosis factor superfamily member 12 (TWEAK) | Cytokine regulating inflammation, cell death and tissue remodelling. |
| *TNR* | Tenascin-R | CNS extracellular matrix glycoprotein involved in neuronal adhesion and perineuronal net formation. |
| *TPR* | Translocated promoter region protein | Nuclear pore basket scaffold involved in mRNA export and mitotic checkpoint regulation. |
| *TREM2* | Triggering receptor expressed on myeloid cells 2 | Microglial surface receptor regulating phagocytosis, lipid metabolism and inflammatory responses. |
| *TUBA1A* | Tubulin α-1A chain | Neuronal α-tubulin isoform required for microtubule assembly and neuronal migration. |
| *TWF2* | Twinfilin-2 | Actin-binding protein modulating filament dynamics and endocytosis. |
| *TYMP* | Thymidine phosphorylase (TP, PD-ECGF) | Nucleotide salvage enzyme converting thymidine to thymine; angiogenic and anti-apoptotic activities. |
| *UBA1* | Ubiquitin-like modifier activating enzyme 1 (E1) | E1 enzyme initiating ubiquitin conjugation cascade for proteostasis. |
| *UBE2K* | Ubiquitin-conjugating enzyme E2 K (UE2K) | E2 enzyme promoting polyubiquitination and proteasomal degradation; implicated in aggregation clearance. |
| *UBE2N* | Ubiquitin-conjugating enzyme E2 N (UBC13) | Catalyzes K63-linked polyubiquitin chains important for DNA repair and NF-κB signalling. |
| *UCHL1* | Ubiquitin carboxyl-terminal hydrolase L1 | Neuronal deubiquitinase involved in ubiquitin recycling and proteostasis. |
| *VASP* | Vasodilator-stimulated phosphoprotein | Actin-regulatory protein promoting filament elongation and focal adhesion dynamics. |
| *VCL* | Vinculin | Focal adhesion protein linking integrins to actin cytoskeleton; mediates mechanotransduction. |
| *VCP* | Valosin-containing protein (p97) | AAA+ ATPase coordinating protein degradation, membrane fusion and autophagy (AAA+ ATPAses are a large superfamily of ATPases characterized by a conserved catalytic module, the AAA+ module). |
| *VEGFC* | Vascular endothelial growth factor C | Growth factor mediating angiogenesis and lymphangiogenesis. |
| *WIF1* | Wnt inhibitory factor 1 | Secreted Wnt antagonist that binds extracellular Wnt ligands to inhibit signalling. |
| *WNT5A* | Wnt family member 5A | Non-canonical Wnt ligand regulating cell polarity, migration and developmental patterning. |
| *XRCC5* | X-ray repair cross-complementing protein 5 (Ku80) | DNA-binding subunit of Ku heterodimer required for non-homologous end-joining. |
| *YWHAB* | 14-3-3 protein β (beta) | Adapter/scaffold protein that binds phospho-serine motifs to regulate signalling and apoptosis. |
| *YWHAQ* | 14-3-3 protein θ (theta) | Adapter protein modulating localization/activity of phosphorylated partners. |
| *YWHAZ* | 14-3-3 protein ζ (zeta) | 14-3-3 family scaffold involved in signal transduction and cell survival. |
