# Supplementary material for: The Nodding syndrome cerebrospinal fluid proteome: a lens into neurodevelopmental failure consistent with environmentally triggered MECP2 dysregulation?
Source: Front Mol Neurosci. 2026 Jan 29;19:1717920. doi: 10.3389/fnmol.2026.1717920 (PMC12894381; doi:10.3389/fnmol.2026.1717920)
Supplement: Supplementary file 4 [file Table_2.docx]

**Supplemental Table 2.** **Comparison of the clinical and neuropathological features of Rett syndrome (MECP2 loss-of-function)** (391-397), **MECP2 Duplication syndrome (MECP2 gain-of-function)** (39,169,172,178,222, 398-403), **and Nodding syndrome** (3,5,7,8,9,18,20,29,171,177,404-406). References are listed in the main reference list.

| CLINICAL FEATURES | RETT SYNDROME  (mostly ♀) | MECP2 DUPLICATION  SYNDROME  (mostly ♂) | NODDING  SYNDROME  (♀, ♂) |
| --- | --- | --- | --- |
| Age at onset | 6-18 months | 9.7y (♂); 10.9y (♀) | 3-18y |
| Regression | Normal development for 6-18 months followed by rapid regression and loss of motor/language skills | Early developmental delay followed by slower or later regression | Progressive physical and cognitive decline follows the onset of head nodding (atonic) and later seizures |
| Life expectancy | Previously fatal by the teenage years. Survival improvement (40-50y) with nutritional support | Only half survive past 25y | Previously fatal by age 25y. Improvement in survival with nutritional support |
| Heterogeneity | High | High | High |
| Growth failure | Pervasive with some cases of microcephaly, small hands/feet | Pervasive, small hands/feet | Pervasive |
| Stunting/wasting | Underweight, decreased muscle mass | Normal nutritional requirements cannot be met leading to unintended malnourishment, stunting, and wasting | Severe malnutrition and avitaminoses, wasted muscles, stunted growth and marasmic-kwashiokor stigmata |
| Communication skills | Absent/limited speech, prelinguistic (i.e., eye contact/gazing) skills | Absent/limited speech, babbling | Absent/limited speech |
| Movement disorders | Failure to walk, dyspraxic wandering, ataxia, dystonia, late motor decline (hypoactive behavior, Parkinsonian features such as rigidity, hypomimia, retropulsion), hand stereotypes (wringing, squeezing, clapping, tapping, rubbing) | Failure to walk, ataxic gait, hypotonia followed by progressive spasticity (lower limbs), choreiform movements, hand stereotypes (flapping, mouthing, clapping, wringing, clasping, biting) | Progressive motor decline, ataxic gait, spasticity, Parkinsonian features, masked facies, very slow word articulation, facial tics when speaking |
| Recurrent infections  (respiratory) | Respiratory infections, aspiration/asphyxiation, respiratory failure | Pneumonia, bronchitis, lung abnormalities, gastric content in the lungs | Pneumonia, measles, lung abnormalities, gastric content in the lungs |
| Other infections | Not reported | Otitis, UTI, sepsis, immunosuppression | Otitis, *O. volvulus, M. perstans, N. americanus,* malaria, other (bacterial, viral, parasitic) infections |
| Gastrointestinal issues | Chewing and swallowing difficulties, bowel function issues, gastroesophageal reflux and constipation | Swallowing difficulties including aspiration and feeding problems, abdominal bloating, air-swallowing, bowel obstruction, constipation, gastro-esophageal reflux | Swallowing difficulties including aspiration and feeding problems, gastro-esophageal reflux |
| Cardiovascular health | Irregular heartbeat, tachycardia, bradycardia, prolonged corrected QT interval | Congenital heart defects | Heart failure reported as comorbidity |
| Epilepsy/seizures | Focal epilepsy more common than generalized epilepsy. Complex partial generalized tonic-clonic, tonic and myoclonic seizures. with absences and clonic seizures being less frequent. Non-epileptic paroxysmal events also common | Epilepsy occurring in > 50% of individuals. Generalized tonic-clonic (grand mal) seizures are common, followed by atonic (head/neck and trunk drop attacks), absence, and myoclonic seizures.  Eating-induced reflex seizures | Epilepsy occurring in 40-50% of individuals. Generalized epilepsy more common than focal epilepsy. Atonic head nodding seizures followed (or not) by generalized tonic-clonic (grand mal), myoclonic, absence, and/or complex-partial seizures. Non-epileptic paroxysmal events associated with fear, panic and visual hallucinations. Eating-induced reflex seizures |
| Seizure triggers | Reflex seizures, triggered by different stimuli (proprioception or eating). | Feeding, infections, fever, emotional upset, sleep deprivation, arousal from sleep, facial tactile stimuli, sudden noise | Sight of food, feeding, cold temperatures, fever, infections, arousal from sleep |
| EEG | Normal before 18 months. Some cases show slowing of the posterior background rhythm. Between 2-10y, focal Rolandic epileptiform abnormalities that evolve to pseudoperiodic delta activity and generalized rhythmic spike discharges (during sleep). After 10y, multifocal and generalized epileptiform abnormalities and rhythmic slow (theta) activity primarily in the frontal-central regions | Abnormal background activity. Generalized slowing with generalized and focal/ multifocal asynchronous discharges and paroxysmal activity, prominent on the frontal areas. | Generalized slow waves (or sharp-and-slow wave complexes) followed by electrodecrement and superimposed gamma activity. Some EEGs show 2-3.5 Hz spike-and-wave discharges intermingled with sharp waves. >50% of interictal abnormalities are hyperventilation-activated. In all ictal EEGs, head nodding episodes came in clusters during hyperventilation. Bilateral frontotemporal or frontocentrotemporal emphasis |
| Musculoskeletal issues | Scoliosis, kyphosis, muscle hypotonia evolving to rigidity, contractures, low bone density and fractures | Scoliosis, kyphosis, pigeon chest (*pectus carinatum*). spina bifida, tapered fingers, flat feet (*pes planus)*, low bone density and fractures | Scoliosis, kyphosis, pigeon chest (*pectus carinatum*), knocked knees (*genu valgum*), flat feet (*pes planus*) and hands due to peripheral muscle wasting, thin cylindrical digits, delayed bone age and osteopenia, flexion limb deformities and contractures around the major joints |
| Urogenital issues | Urological dysfunction including urinary incontinence, frequent UTIs, kidney stones and urine retention | Vesicoureteral reflux, bladder hypertrophy, hydronephrosis, pyelonephritis, kidney stones, ureteral dilation, urinary retention, micropenis, cryptorchidism | Urinary incontinence, delayed puberty |
| Unusual eye movements and/or vision difficulties | Intense staring, blinking, crossed eyes, closing one eye at a time | Divergent strabismus (exotropia), likely related to visual impairments such hyperopia and amblyopia | NS-unrelated onchocerciasis or “river blindness” |
| Hearing impairment | Sensorineural hearing loss, conductive and mixed hearing loss | Conductive hearing loss due to otitis media | Reported hearing loss and deafness |
| Clinical overlap with autism spectrum disorders (ASD) | Autistic traits, especially during the regression period | Traits akin to idiopathic ASD (i.e., social affect, restricted/repetitive behaviors) | Clinical, biochemical and behavioral similarities with ASD |
| Behavioral/mood disorders | Irritability, crying, screaming for hours, fear and anxiety, wandering | Aggression/temper tantrums, attention issues, high pain tolerance, depression, hyperactivity, poor sense of danger | Aggressive outbursts, attention issues, major depression, hyperactivity, poor sense of danger, wandering and running |
| Autonomic issues | Irregular breathing, breath holding, hyperventilation, blowing out air or saliva, drooling, swallowing air, cold hands and feet, decreased response to pain, sudden unexpected death | Breath holding, hyperventilation, vasomotor issues, livedo of the limbs, cold hands and feet that periodically sweat, problems regulating body temperature, hypersalivation and drooling | Hyperventilation, cold hands and feet, problems regulating body temperature, hypersalivation and drooling |
| Sleep problems | Sleep and daytime apnea, irregular sleep, hypersomnia or somnolence during the day, nocturnal awakening crying or screaming | Sleep apnea, irregular sleep, hypersomnia or somnolence during the day, nocturnal awakening, sleep-wake rhythm disorder | Hypersomnia/somnolence during the day, nocturnal awakening |
| Mental retardation | Severe, loss of intellectual functioning | Severe mental retardation with progressive neurological symptoms | Different degrees of mental retardation. Severe with loss of intellectual functioning and progressive neurological symptoms |
| Facial dysmorphia | Few unusual facial features | Facial hypotonia, hypomimia, midface hypoplasia, short nose with depressed nasal bridge, large and/or low-set ears, deep-set eyes, hypertelorism, down-slanting palpebral fissures, epicanthal folds, small mouth/open appearance, tented upper lip, deciduous teeth, undersized jaw (micrognathia) | Expressionless face or “myopathic facies”, enlarged lower lip, open mouth appearance, flat bridged nose, malformed teeth |
| NEUROPATHOLOGY | **RETT SYNDROME**  (mostly ♀) | **MECP2-DUPLICATION**  **AND TRIPLICATION**  **SYNDROMES**  (mostly ♂) | **NODDING**  **SYNDROME**  **(**♀, ♂) |
|  | Smaller brain (smaller head). No gross abnormalities, gliosis, or neuron-migration defects. Decreased synaptic density, decreased dendritic arborization and increased neuronal cell packing in the hippocampus. No signs of cell loss or neurodegeneration | Cerebral cortical and cerebellar (Purkinje cell) degeneration. Hippocampal (CA1) pathology prominent in a mouse transgenic line (MECP2-TG3) expressing MECP2 at levels that are 3–5 times higher than those observed in wild-type mice. In this model, mirroring patients with MECP2-Triplication Syndrome, neuronal loss in the cortex and hippocampus is preceded by upregulation of glial fibrillary acidic protein and tau, suggesting that brain disease results from astrocyte dysfunction that leads to tau-mediated, MK801-blockable excitotoxic neurodegeneration. | Tau pathology (tau positive deposits in the form of neurofibrillary tangles, pre-tangles and dot-like grains and threads in the neuropil) with cerebellar and white matter degeneration. Some degree of tau pathology in the neocortex and in the locus ceruleus with frequent involvement of the substantia nigra and tegmental nuclei. Lesser involvement of other grey matter sites. Lack of glial tau pathology. Tau pathology in the neocortex shows a multifocal superficial laminar pattern.  Other authors emphasize neuroinflammation and no generalized tauopathy.  Cerebellar atrophy and loss of Purkinje cells with hyperplasia of the Bergmann glia, gliosis and CD68-positive macrophage clusters in various degrees. Immunohistochemistry for amyloid β, α-synuclein, or TDP-43 was negative. Mild to sparse AT8-positive neurofibrillary tangle-like structures and threads preferentially in the frontal and parietal cortex, thalamic and hypothalamic regions, mesencephalon and corpus callosum |
